# ZBTB21 Is a Dual Suppressor of Pyroptosis and MHC‐I Antigen Presentation That Promotes Tumor Immune Evasion

**DOI:** 10.1002/advs.202519836

**Published:** 2026-02-08

**Authors:** Lei Zhao, Linlin Sheng, Jianhao Qiu, Junjie Ma, Kai Jin, Binggong Zhao, Tianrun Miao, Jing Chen, Yehong Li, Zhan Zhang, Dongfeng Sun, Yongmeng Li, Hui Tian

**Affiliations:** ^1^ Department of Thoracic Surgery Shandong Key Laboratory of Digital Diagnosis and Treatment of Thoracic Oncology Shandong Engineering Research Center of Intelligent Surgery The First Affiliated Hospital of Shandong First Medical University & Shandong Provincial Qianfoshan Hospital Jinan China; ^2^ Department of Thoracic Surgery Qilu Hospital of Shandong University Jinan Shandong China; ^3^ College of Chinese Medicine Shandong University of Traditional Chinese Medicine Jinan China; ^4^ School of Pharmaceutical Sciences Key Laboratory of Bioorganic Phosphorus Chemistry and Chemical Biology (Ministry of Education) Tsinghua University Beijing China

**Keywords:** immune checkpoint blockade, MHC‐I, pyroptosis, tumor immune evasion, ZBTB21

## Abstract

Immune checkpoint blockade (ICB) efficacy is limited by tumor‐intrinsic immune escape mechanisms. This study identifies the transcription factor ZBTB21 as a central orchestrator of dual immunosuppressive programs. ZBTB21 epigenetically silences gasdermin D (GSDMD)‐dependent pyroptosis by restricting STAT1‐mediated chromatin accessibility via H3K27ac modulation at the GSDMD locus. Simultaneously, it represses MHC‐I antigen presentation by attenuating IRF1 expression and its transactivation capacity. Genetic ablation of ZBTB21 unleashes pyroptotic cell death and enhances tumor antigen presentation, establishing a self‐reinforcing cycle that recruits and activates CD8^+^ T cells. This dual activation overcomes ICB resistance in murine models, while B2M deletion ablates efficacy, confirming MHC‐I dependency. Pharmacological inhibition of ZBTB21 with dobutamine disrupts its DNA‐binding domain, which triggers pyroptotic inflammation and MHC‐I upregulation to synergize with anti‐PD‐1 therapy. Thus, ZBTB21 represents a druggable nexus coordinating pyroptosis resistance and antigen presentation escape, providing a combinatorial strategy to reinvigorate antitumor immunity.

## Graphical abstract

ZBTB21 is a transcription factor that epigenetically suppresses pyroptosis and MHC‐I antigen presentation, enabling tumor immune evasion. Genetic ablation of ZBTB21 activates pyroptotic cell death and enhances antigen presentation, recruiting CD8^+^ T cells to overcome immune checkpoint blockade resistance. Pharmacological inhibition with dobutamine disrupts ZBTB21 function and synergizes with anti‐PD‐1 therapy.

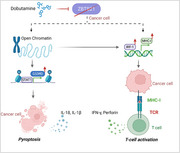

## 1 Introduction

Immune checkpoint inhibitors (ICIs), particularly PD‐1/PD‐L1‐targeting monoclonal antibodies, represent a landmark breakthrough in cancer therapy. However, their efficacy remains constrained by low objective response rates (<30%) in solid tumors and frequent resistance development, collectively limiting long‐term survival benefits [1, 2]. These limitations primarily stem from two interconnected barriers: tumor‐intrinsic immune evasion (e.g., MHC‐I‐mediated antigen presentation defects) and immunosuppressive tumor microenvironment (TME) [3, 4, 5].

In antigen presentation escape, downregulated MHC‐I expression impairs CD8^+^ T cell recognition, promoting primary/acquired ICIs resistance [6, 7, 8, 9, 10]. Concurrently, insufficient T cell infiltration in “cold tumors” restricts immune efficacy [3, 4, 5]. Emerging evidence indicates that pyroptosis—Gasdermin‐mediated inflammatory cell death—converts “cold” to “hot” tumors by releasing DAMPs and cytokines (e.g., IL‐1β/IL‐18) that recruit antigen‐presenting cells and drive T cell influx [11, 12, 13, 14, 15].

Effective antitumor immunity requires coordination of the “Cancer‐Immunity Cycle” [16, 17, 18, 19]: pyroptosis‐driven T cell recruitment (priming) must synergize with MHC‐I‐dependent antigen recognition (effector killing). Notably, isolated pyroptosis induction is ineffective when MHC‐I defects impair cytotoxicity; conversely, enhanced antigen presentation fails without sufficient T cell infiltration.

To overcome these barriers, we propose a synergistic strategy: co‐activating tumor cell pyroptosis to amplify immune priming while enhancing MHC‐I antigen presentation to optimize effector killing. Here, we identify the transcription factor ZBTB21 as the master epigenetic regulator coordinating both evasion mechanisms.

We demonstrate that ZBTB21 epigenetically represses GSDMD‐dependent pyroptosis via STAT1‐driven chromatin restriction through H3K27ac hypomodification at the GSDMD locus, suppressing inflammasome activation. Concomitantly, it attenuates MHC‐I antigen presentation by inhibiting IRF1 transcription and transactivation capacity. Notably, genetic ablation of ZBTB21 unleashes pyroptotic death and enhances MHC‐I expression, establishing an immunogenic cycle that recruits CD8^+^ T cells and licenses tumor cell killing. This dual activation overcomes resistance to PD‐1/CTLA‐4 blockade and therapeutic vaccination, with B2M‐knockout confirming strict MHC‐I dependency. Pharmacologically, dobutamine‐mediated ZBTB21 inhibition—via zinc‐finger DNA‐binding disruption—recapitulates pyroptosis and MHC‐I upregulation, synergizing with anti‐PD‐1 therapy to eradicate tumors. Collectively, ZBTB21 represents a druggable nexus integrating pyroptosis resistance and antigen presentation escape, providing a combinatorial blueprint to potentiate antitumor immunity.

## 2 Result

### 2.1 ZBTB21 Suppresses Tumor‐Intrinsic Pyroptosis to Drive Tumor Progression Through Regulation of the GSDMD

**FIGURE 1:**
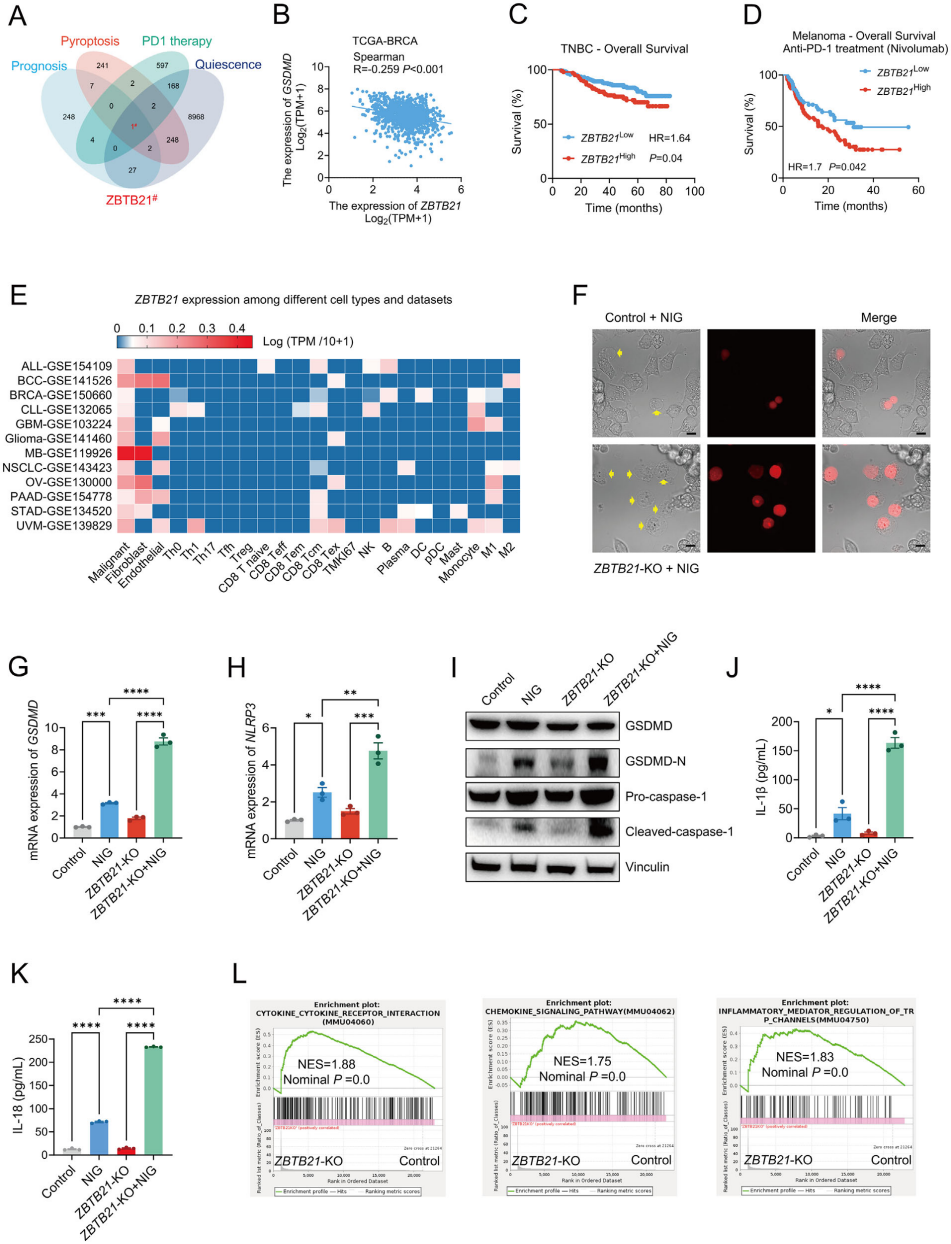
ZBTB21 deficiency activates pyroptosis through the NLRP3/GSDMD axis to enhance immunotherapy response. (A) Schematic description of the selection of ZBTB21 from multiple databases: Prognosis: TCGA (hazard ratio > 1.5, *p* < 0.05); refractory to PD‐1 blockade: GEO: GSE78220 (*p* < 0.05; |log2fold change (FC)| > 1.2); T Cell State Score (Quiescence): TCGA (Pearson's R > 0.1; *p* < 0.05); Pyroptosis related transcription factors: ChIP‐Atlas, KnockTF, and JASPAR databases. (B) Negative correlation between *ZBTB21* and *GSDMD*‐mRNA expression levels in tumor samples from TCGA‐BRCA dataset. (C) Kaplan‐Meier overall survival curves of TNBC patients with *ZBTB21* ^low^ (*n* = 621) versus *ZBTB21* ^high^ (*n* = 304). *ZBTB21*‐mRNA expression of the auto‐selected best cutoff. (D) Kaplan–Meier overall survival curves of Melanoma patients receiving anti‐PD‐1 treatment with *ZBTB21* ^low^ (*n* = 123) versus *ZBTB21* ^high^ (*n* = 155). *ZBTB21*‐mRNA expression of the auto‐selected best cutoff. (E) Analysis of *ZBTB21* gene expression across diverse cell types within various tumor using the pan‐cancer single‐cell sequencing data. (F) The images show representative micrographs of pyroptosis induced by Nigericin (NIG), where pyroptotic cells are stained red with propidium iodide (PI). Yellow arrows indicate key pyroptotic features. Scale bar: 20 µm. (G,H) Relative levels of *GSDMD* and *NLRP3*‐mRNA expression measured by qPCR (*n* = 3). (I) Immunoblotting results showing the expression of the indicated proteins in *ZBTB21*‐KO and Control 4T1 cells treated with or without NIG. (J,K) IL‐1β and IL‐18 concentration in supernatant of *ZBTB21*‐KO and Control 4T1 cells treated with or without NIG (*n* = 3). (L) Transcriptomic analysis of *ZBTB21*‐KO versus Control 4T1 cells. GSEA plots corresponding to each Hallmark pathway, displaying the normalized enrichment score (NES), nominal *p* value (Nominal P), and FDR (FDR q). Significantly enriched pathways were defined as those with |NES| > 1, nominal *p* value < 0.05, and FDR q value < 0.25. Data are presented as mean ± SEM. ^*^ *p* < 0.05; ^**^ *p* < 0.01; ^***^ *p* < 0.001; ^****^ *p* < 0.0001. Data were analyzed by one‐way ANOVA, Log‐rank (Mantel‐Cox) test or Spearman test.

Our gene selection strategy employed a multidimensional approach integrating clinical prognosis, molecular mechanism, therapeutic response, and immune microenvironment characteristics. First, we systematically analyzed TCGA transcriptomic data from multiple cancers to identify genes whose elevated expression significantly correlated with poor patient survival, reflecting their potential critical roles in tumor progression. Second, through multiple databases (ChIP‐Atlas [20], KnockTF [21], and JASPAR [22] databases). Screening and chromatin immunoprecipitation sequencing motif analysis, we focused on transcription factors potentially regulating pyroptosis executors (GSDM family), effectors (CASP1/3/4/5/8/11), and immune checkpoint components (IFNGR1), given pyroptosis's critical role in both antitumor immunity and immunosuppressive microenvironment formation. Third, leveraging RNA‐seq data from PD‐1 blockade‐treated cohorts [23], we identified differentially expressed genes (DEGs) between responders and non‐responders, prioritizing candidates potentially involved in alternative immune resistance pathways. Finally, through TCellSI (T Cell State Identifier) [24], we selected genes positively correlated with T cell quiescence, hypothesizing these might participate in maintaining immune‐suppressive niches. The intersection of these four stringent criteria revealed ZBTB21 as a prime candidate (Figure 1A), whose zinc finger structure suggests transcriptional regulatory capacity, while its clinical association pattern implies potential involvement in establishing treatment‐refractory tumor states through immune‐modulatory mechanisms. This combinatorial strategy ensures both mechanistic relevance and clinical translatability of identified targets.

Initial analysis of the TCGA dataset revealed a significant mutually exclusive expression pattern between ZBTB21 and GSDMD in breast invasive carcinoma (BRCA) (Figure 1B), with this reciprocal relationship being consistently observed in other tumor types, including bladder urothelial carcinoma (BLCA), cervical squamous cell carcinoma and endocervical adenocarcinoma (CESC), colon adenocarcinoma (COAD), and rectum adenocarcinoma (READ) (Figure S1A). In contrast, no significant negative correlation was observed between ZBTB21 and GSDMA, GSDMB, GSDMC, or GSDME (Figure S1B). Notably, elevated ZBTB21 expression demonstrated strong clinical correlation with diminished overall survival rates in patients (Figure 1C). To further validate the independent prognostic value of ZBTB21, a multivariable Cox proportional hazards model was employed, adjusting for key clinicopathological covariates including pathological T stage, patient age, and molecular subtypes (PR, ER, and HER2 status). The analysis confirmed that high ZBTB21 expression remained significantly associated with poorer overall survival (hazard ratio [HR] = 1.467, 95% confidence interval [CI]: 0.919–2.342, *p* < 0.001), establishing it as an independent risk factor (Figure S1C). Diagnostic evaluation using ROC curve analysis further revealed robust discriminatory capacity of ZBTB21 in distinguishing cancer from normal tissues across multiple tumor types (Figure S1D). This prognostic association was further extended to immunotherapy outcomes, where transcriptomic profiling of anti‐PD‐1 (Nivolumab) ‐treated cohorts revealed significantly poorer therapeutic responses in patients with high ZBTB21 expression (Figure 1D). The dual clinical relevance of ZBTB21 in both disease progression and immune checkpoint blockade resistance prompted a systematic investigation into its cellular origin. Single‐cell RNA sequencing analysis [25] of various human specimens consistently identified malignant cells as the predominant source of ZBTB21 expression (Figure 1E). This tumor cell‐specific expression pattern, coupled with ZBTB21's structural features, including conserved zinc‐finger domains [26], suggests its potential role in modulating tumor‐intrinsic pathways that may influence both oncogenic progression and immune evasion mechanisms.

To investigate the functional impact of ZBTB21 loss on pyroptosis‐mediated antitumor immunity, we generated ZBTB21‐knockout (*ZBTB21*‐KO) 4T1 cells using CRISPR‐Cas9 (Figure S2A,B). Nigericin (NIG)‐induced pyroptosis in *ZBTB21*‐KO cells was evidenced by positive propidium iodide (PI) staining, indicative of plasma membrane permeabilization, along with the characteristic morphological features [27] of cellular swelling and dynamic bubble‐like protrusions (Figure 1F). Anti‐GSDMD‐N immunofluorescence confirmed aggregation of N‐terminal fragment (GSDMD‐N) on the plasma membrane of NIG‐stimulated cells (Figure S2C). Strikingly, ZBTB21 ablation selectively upregulated *GSDMD* mRNA levels without altering expression of other gasdermin family members (*GSDMA*, *GSDMC*, or *GSDME*; Figure 1G; Figure S2D). The results validated the mutually exclusive relationship between ZBTB21 and GSDMD identified by bioinformatic analyses (Figure 1B; Figures S1B). Consistent with NLRP3 inflammasome‐mediated cleavage during pyroptosis [11], quantitative analysis demonstrated significant elevation of *NLRP3* mRNA in *ZBTB21*‐KO cells (Figure 1H). Immunoblotting confirmed increased accumulation of the proteolytically activated GSDMD‐N of GSDMD and activated caspase‐1 (Figure 1I), and *ZBTB21*‐KO cells secreted significantly higher levels of IL‐1β (Figure 1J) and IL‐18 (Figure 1K) upon pyroptosis induction. Whole‐transcriptome analysis revealed 1168 DEGs with enrichment in immune‐activating pathways, including cytokine‐cytokine receptor interaction and chemokine signaling (Figure 1L). Importantly, ZBTB21 depletion did not alter baseline proliferation rates, migration, or apoptosis susceptibility (Figure S2E–G), indicating that it specifically regulates inflammasome‐driven pyroptosis.

### 2.2 ZBTB21 Loss Enhances CD8^+^ T Cell‐Mediated Anti‐Tumor Immunity in a GSDMD‐Dependent Manner

**FIGURE 2:**
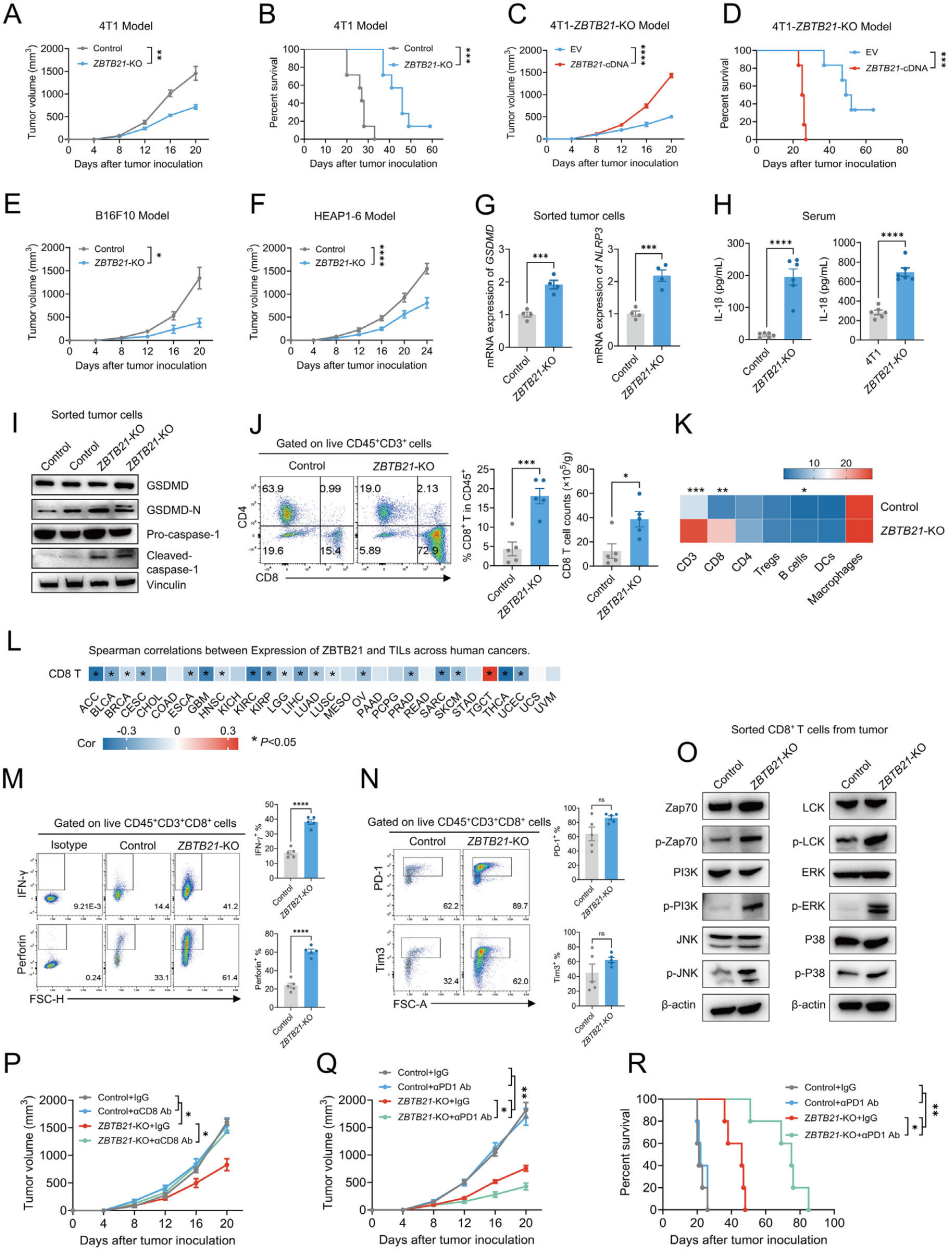
ZBTB21 loss activates GSDMD pyroptosis to enhance CD8^+^ T cell immunity and amplify checkpoint blockade. (A,B) Tumor growth and overall survival curves for 4T1 Control and *ZBTB21*‐KO tumor‐bearing mice (*n* = 7). (C,D) Tumor growth and overall survival curves for tumor‐bearing mice implanted with *ZBTB21*‐KO cells rescued by *ZBTB21‐*cDNA or empty vector (EV) (*n* = 6). (E,F) Tumor growth curves for B16F10 (*n* = 6) or HEPA1‐6 (*n* = 7) Control and *ZBTB21*‐KO tumor‐bearing mice. (G) Relative levels of *GSDMD* and *NLRP3*‐mRNA expression measured by qPCR in tumor cells (CD45^−^ CD31^−^ CD90.2^−^ cells) isolated from 4T1 Control or *ZBTB21*‐KO tumor‐bearing mice (*n* = 4). (H) IL‐1β and IL‐18 concentration in serum from 4T1 Control or *ZBTB21*‐KO tumor‐bearing mice (*n* = 6). (I) Immunoblotting results showing the expression of the indicated proteins of tumor cells isolated from 4T1 Control or *ZBTB21*‐KO tumor‐bearing mice (*n* = 2). (J) Representative staining profiles (left) of CD4/CD8 and percentages (right) of CD8^+^ T cells among CD45^+^ cells in 4T1 Control or *ZBTB21*‐KO tumors (*n* = 5). (K) Heatmap based on percentages of the indicated immune cell populations among CD45^+^ cells in 4T1 Control or *ZBTB21*‐KO tumors (*n* = 4). (L) Association between *ZBTB21* expression and CD8^+^ T cells infiltration across human cancers (TCGA dataset). (M) Representative staining profiles (left) and percentages (right) of IFN‐γ and Perforin expression in tumor‐infiltrating CD8^+^ T cells from 4T1 Control and *ZBTB21*‐KO tumors (*n* = 5). (N) Representative staining profiles (left) and percentages (right) of PD‐1 and Tim3 expression in tumor‐infiltrating CD8^+^ T cells from 4T1 Control and *ZBTB21*‐KO tumors (*n* = 5). (O) Immunoblotting results for T cell activation signaling pathway‐related protein phosphorylation in tumor‐infiltrating CD8^+^ T cells isolated from 4T1 Control or *ZBTB21*‐KO tumor‐bearing mice. (P) Tumor growth curves for 4T1 Control or *ZBTB21*‐KO tumor‐bearing mice treated with αCD8 or IgG antibodies (*n* = 5). (Q,R) Tumor growth and overall survival curves for 4T1 Control or *ZBTB21*‐KO tumor‐bearing mice treated with αPD1 or IgG antibodies (*n* = 5). Data are presented as mean ± SEM. ^*^ *p* < 0.05; ^**^ *p* < 0.01; ^***^ *p* < 0.001; ^****^ *p* < 0.0001. Data were analyzed by two‐way ANOVA, Log‐rank (Mantel‐Cox) test, unpaired two‐tailed Student's *t*‐test or Spearman test.

To interrogate the immunomodulatory role of ZBTB21 in vivo, we established orthotopic mammary tumors in immunocompetent Balb/c mice using *ZBTB21*‐KO cells. Remarkably, ZBTB21 deficiency significantly suppressed tumor growth, resulting in reduced tumor size and increased animal survival (Figure 2A,B; Figure S3A). This tumor suppressive phenotype was ZBTB21 dependent, as reintroduction of ZBTB21 cDNA into *ZBTB21*‐KO cells restored aggressive growth and mortality rates (Figure 2C,D; Figure S3B). The broad relevance of this mechanism was confirmed across multiple allogeneic models—including B16F10 melanoma and Hepa1‐6 hepatocellular carcinoma—where ZBTB21 loss universally suppressed tumor growth (Figure 2E,F; Figure S3C–H). Critically, this antitumor effect was immunity dependent, as ZBTB21‐deficient and wild‐type cells exhibited comparable proliferation rates in vitro (Figure S2E) and formed equally lethal tumors in NCG immunodeficient mice (Figure S3I). Notably, consistent with in vitro findings, *ZBTB21*‐KO tumors demonstrated upregulated GSDMD and NLRP3 expression in tumor cells relative to controls (Figure 2G). The serum levels of IL‐1β and IL‐18 in *ZBTB21*‐KO mice were also significantly increased (Figure 2H), accompanied by prominent accumulation of the pyroptosis‐executing GSDMD‐N fragment and activated caspase‐1 (Figure 2I).

Immune profiling revealed substantial remodeling of the TME, characterized by expanded CD8^+^ T cells (Figure 2J,K; Figure S4A), alongside reduced M2‐polarized macrophages (Figure S4B). TCGA data analysis corroborated these findings, demonstrating a negative correlation between ZBTB21 expression and CD8^+^ T cell infiltration across most cancer types analyzed (Figure 2L; Figure S4C). Notably, tumor‐infiltrating CD8^+^ T cells isolated from *ZBTB21*‐KO tumors exhibited enhanced functional capacity evidenced by elevated IFN‐γ secretion and increased perforin production (Figure 2M), without alterations in exhaustion markers (PD‐1, TIM‐3; Figure 2N). Mechanistically, Western blot analysis revealed significantly heightened phosphorylation levels of key signaling molecules ZAP70, PI3K, JNK, LCK, ERK1/2, and p38 in CD8^+^ T cells from *ZBTB21*‐KO tumors compared to those from control 4T1 tumors (Figure 2O).

Depletion studies established CD8^+^ T cells as indispensable mediators of this response: anti‐CD8α treatment fully abrogated the growth inhibition of *ZBTB21*‐KO tumors (Figure 2P; Figure S4D,E), while B cell and macrophage depletion had no effect (Figure S4F–I). Furthermore, ZBTB21 deficiency markedly enhanced the antitumor efficacy of anti‐PD1 monoclonal antibody (Figure 2Q,R). Collectively, these findings demonstrate that ZBTB21 deficiency triggers GSDMD‐dependent pyroptotic signaling in tumor cells, which remodels the tumor immune microenvironment to potentiate CD8^+^ T cell infiltration and cytotoxic function. This pyroptosis‐driven “cold‐to‐hot” transformation is evidenced by enhanced TCR‐proximal signaling (ZAP70/LCK) and downstream MAPK pathway activation in the infiltrating CD8^+^ T cells. This immunomodulatory mechanism not only underlies intrinsic tumor suppression but also synergizes with PD‐1 blockade therapy, positioning ZBTB21 as a critical regulator of antitumor immunity. To definitively establish the functional requirement of GSDMD in ZBTB21 deficiency induced antitumor immunity, we administered the GSDMD‐specific inhibitor Necrosulfonamide (iGSDMD) to tumor‐bearing mice with ZBTB21 knockout. GSDMD inhibition significantly reversed the tumor growth suppression and the increase in CD8^+^ T cell infiltration mediated by *ZBTB21*‐KO, directly demonstrating that GSDMD‐dependent pyroptosis is essential for the ZBTB21 regulated “cold‐to‐hot” tumor transition (Figure S4J).

The aforementioned results demonstrate that ZBTB21 deficiency remodels the tumor immune microenvironment (TME) by inducing tumor cell pyroptosis, thereby successfully recruiting and enhancing the antitumor activity of infiltrating CD8^+^ T cells. However, effective antitumor immunity depends not only on the presence and functional activation of effector CD8^+^ T cells but also critically on their ability to precisely recognize tumor cell surface antigen peptides presented by MHC‐I molecules via the T cell receptor. Therefore, a key question arises: Within the context of the pyroptosis‐driven inflammatory TME induced by ZBTB21 deficiency, does ZBTB21 loss itself enhance tumor cell antigen processing and presentation capacity, particularly the MHC‐I dependent pathway, thereby synergistically optimizing effector CD8^+^ T cell recognition and killing of tumor cells?

### 2.3 ZBTB21 Deficiency Enhances Tumor Antigen Presentation and CD8^+^ T Cell Recognition

**FIGURE 3:**
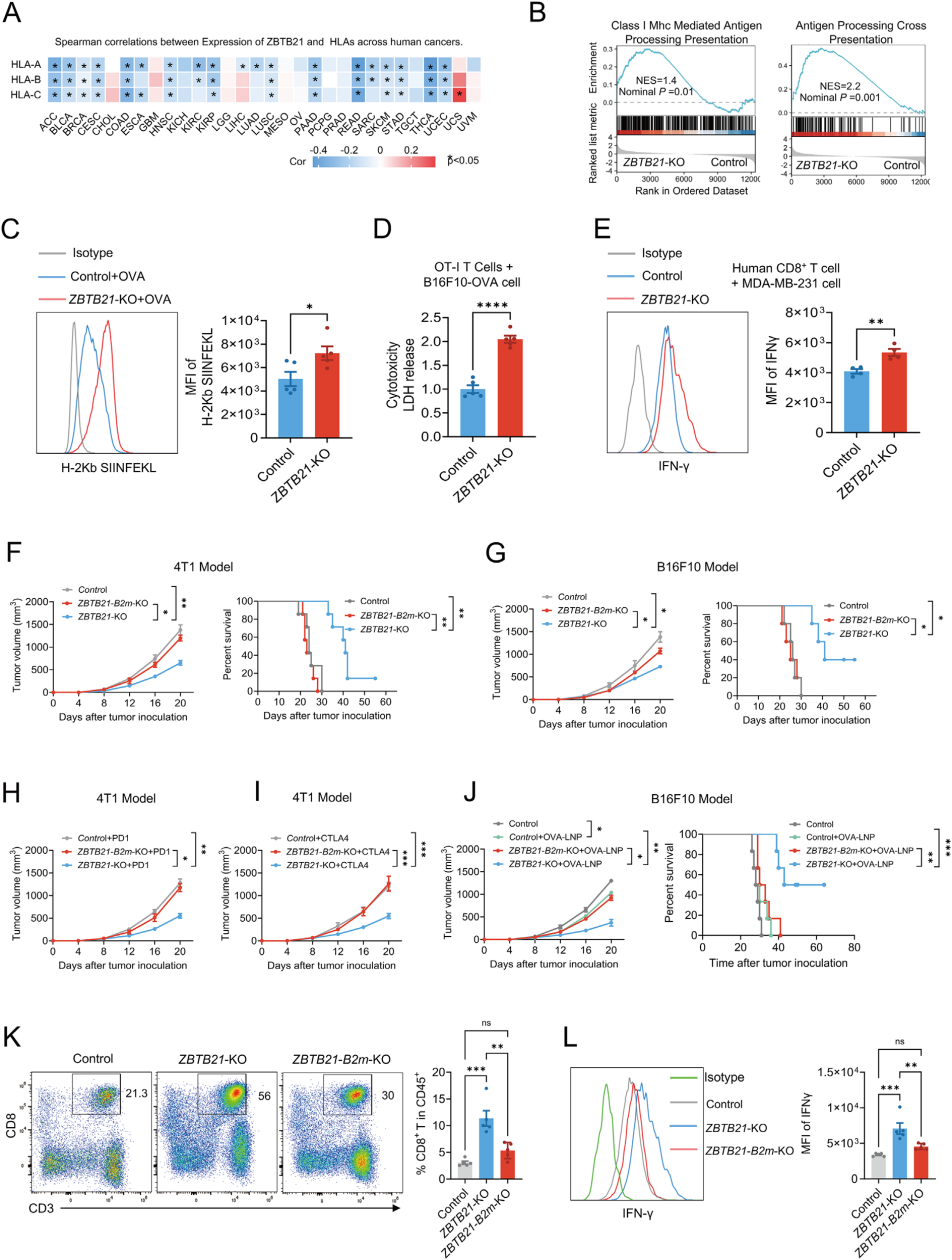
ZBTB21 loss enhances tumor immunogenicity and response to immunotherapy through MHC‐I upregulation. (A) Association between *ZBTB21* expression and HLA/B/C across human cancers (TCGA dataset). (B) Transcriptomic analysis of *ZBTB21*‐KO versus Control 4T1 cells. GSEA plots corresponding to each Hallmark pathway, displaying the normalized enrichment score (NES), nominal *p* value (Nominal P), and FDR (FDR q). A Hallmark pathway was considered significantly enriched if it met the following criteria: |NES| > 1, nominal *p* value < 0.05, and FDR q value < 0.25. (C) Representative staining profiles (left) and H‐2Kb SIINFEKL MFI (right) in Control+OVA and *ZBTB21*‐KO+OVA cells (*n* = 5). (D) Cytotoxicity analysis of splenic OT‐I T cells cocultured with OVA‐expressing B16F10 tumor cells versus *ZBTB21*‐KO B16F10 tumor cells (*n* = 5). (E) Representative staining profiles (left) and IFN‐γ detection results (right) of T cells following co‐culture with *ZBTB21*‐KO MDA‐MB‐231 cells or parental MDA‐MB‐231 cells (*n* = 4). (F,G) Tumor growth curves (*n* = 5) and overall survival curves (*n* = 7) of tumor‐bearing mice inoculated with Control, *ZBTB21*‐*B2m*‐KO, or *ZBTB21*‐KO tumor cells. (H,I) Tumor growth curves for Control, *ZBTB21*‐*B2m*‐KO, or *ZBTB21*‐KO tumor tumor‐bearing mice treated with PD1 or CTLA‐4 antibodies (*n* = 5). (J) Tumor growth (*n* = 5) and overall survival (*n* = 6) curves for Control, *ZBTB21*‐*B2m*‐KO, or *ZBTB21*‐KO tumor‐bearing mice treated with OVA‐encoding mRNA‐LNP vaccine or empty‐vector mRNA‐LNP as control. (K) Representative staining profiles (left) of CD8 and percentages (right) of CD8^+^ T cells among CD45^+^ cells in Control, *ZBTB21*‐*B2m*‐KO, or *ZBTB21*‐KO tumors (*n* = 5). (L) Representative staining profiles (left) and IFN‐γ MFI (right) in Control, *ZBTB21*‐*B2m*‐KO, or *ZBTB21*‐KO tumors (*n* = 5). Data are presented as mean ± SEM. ^*^ *p* < 0.05; ^**^ *p* < 0.01; ^***^ *p* < 0.001; ns, no significance. Data were analyzed by two‐way ANOVA, Log‐rank (Mantel‐Cox) test or unpaired two‐tailed Student's *t*‐test.

TCGA data analysis further corroborated our findings, revealing a significant negative correlation between ZBTB21 expression and classical MHC‐I molecules (HLA‐A/B/C) across most cancer types analyzed (Figure 3A). To further validate this inverse relationship at the protein level and assess its clinical impact, we performed immunohistochemical analysis on clinical specimens. Examination of tumor tissues from 15 patients with lung adenocarcinoma confirmed a significant co‐occurrence of high ZBTB21 protein expression with low MHC‐I protein levels (Figure S5A). Moreover, analysis of a clinical cohort receiving anti‐PD‐1 therapy demonstrated that high tumor ZBTB21 expression correlated with poorer treatment response, whereas high MHC‐I expression predicted superior clinical benefit (Figure S5B–D), underscoring the clinical relevance of the ZBTB21‐MHC‐I axis.

To investigate the impact of ZBTB21 deficiency on the MHC‐I pathway at the cellular level, we performed RNA‐seq on Control and *ZBTB21*‐KO cells. GSEA demonstrated that pathways involved in antigen processing and MHC‐I presentation were significantly enriched and upregulated following ZBTB21 loss (Figure 3B). Specifically, mRNA expression levels of key MHC‐I antigen processing and presentation molecules, including MHC‐I heavy chains (*HLA‐A/B/C*) and antigen peptide transporters (*TAP1/TAP2*), were significantly elevated in *ZBTB21*‐KO cells (Figure S5E,F). Consistent with this, flow cytometric analysis of tumor cells isolated from tumor‐bearing mice confirmed that ZBTB21 knockout effectively upregulated cell surface MHC‐I protein expression (Figure S5G). Given that IFN‐γ is a key cytokine inducing MHC‐I expression [28], we further evaluated the regulatory role of ZBTB21 deficiency on MHC‐I expression under IFN‐γ stimulation. Flow cytometry revealed that exogenous IFN‐γ stimulation significantly enhanced cell surface MHC‐I levels in ZBTB21 deficient cells (Figure S5H). Collectively, these in vitro and in vivo results demonstrate that ZBTB21 ablation effectively enhances MHC‐I molecule expression in tumor cells.

To determine whether ZBTB21 directly influences tumor cell antigen presentation by regulating MHC‐I expression, we employed the classical OVA/OT‐I model system. Control and *ZBTB21*‐KO cells were stably transduced to express ovalbumin (OVA) (designated Control+OVA and *ZBTB21*‐KO+OVA cells, respectively). To assess the level of OVA peptide MHC‐I complexes on the cell surface, we stained cells with a monoclonal antibody specifically recognizing the SIINFEKL immunodominant epitope presented by H‐2Kb. The results showed a significant increase in SIINFEKL/H‐2Kb complex‐specific fluorescence signal on *ZBTB21*‐KO+OVA cells compared to Control+OVA cells (Figure 3C), directly demonstrating that ZBTB21 deficiency increases the abundance of antigen peptide‐loaded MHC‐I complexes on the tumor cell surface.

We next assessed the biological impact of this enhanced antigen presentation on CD8^+^ T cell function. Using an in vitro co‐culture system with CD8^+^ T cells isolated from OT‐I transgenic mouse spleens and OVA‐expressing B16F10 cells (B16F10‐OVA), cytotoxicity assays demonstrated that CD8^+^ T cells exhibited significantly higher killing efficiency against *ZBTB21*‐KO+OVA cells compared to Control+OVA cells (Figure 3D). Furthermore, in a humanized system, co‐culture of human breast cancer cells MDA‐MB‐231 (Control or *ZBTB21*‐KO) with human peripheral blood‐derived T cells revealed that T cells co‐cultured with *ZBTB21*‐KO MDA‐MB‐231 cells secreted significantly higher levels of the activation marker IFN‐γ (Figure 3E). To further validate the clinical relevance across cancer types, we examined human melanoma (A375) and non‐small cell lung cancer (H1299) models. ZBTB21 deletion significantly upregulated MHC‐I expression in both models. Crucially, upon co‐culture with human T cells, ZBTB21‐deficient A375 and H1299 cells potently enhanced T cell activation, as evidenced by increased IFN‐γ production (Figure S5I–L). These results collectively indicate that ZBTB21 deficiency not only enhances tumor cell antigen presentation but also effectively promotes T cell activation and effector function, potentially triggering more potent immune‐mediated tumor cell clearance.

Beta‐2‐microglobulin (B2M) is an essential component required for the stable cell surface expression of all classical MHC‐I molecules [29]. Its deficiency leads to complete loss of MHC‐I surface expression, enabling tumor cells to evade immune recognition, particularly by CD8^+^ T cells [30]. To directly investigate whether ZBTB21 mediates tumor immune evasion through the MHC‐I pathway, we generated double‐knockout cell lines lacking both ZBTB21 and B2m (*ZBTB21*‐*B2m*‐KO) in two murine tumor cell lines (Figure S5M). The double knockout did not affect cell proliferation (Figure S5N). In tumor‐bearing mouse models, *B2m* gene deletion significantly reversed the tumor growth inhibition mediated by ZBTB21 single knockout (*ZBTB21*‐KO) (Figure 3F,G; Figure S6A,B), providing compelling evidence that the antitumor effect of ZBTB21 deficiency depends on an intact MHC‐I antigen presentation pathway.

To further exclude potential compensatory activity of NK cells in the context of MHC‐I deficiency, we performed cell depletion experiments in *ZBTB21‐B2m* double knockout (*ZBTB21*‐*B2m*‐KO) tumor‐bearing models. Results demonstrated that the antitumor effect of ZBTB21 knockout remained strictly and specifically dependent on CD8^+^ T cells, with no compensatory activity observed from NK cells (Figure S6C), confirming that the immune response is mediated exclusively by CD8^+^ T cells rather than innate immune compensation. Next, to functionally validate the irreplaceable role of MHC‐I, we re‐expressed B2m in *ZBTB21*‐*B2m*‐KO cells. This rescue experiment revealed that B2m re‐expression significantly suppressed tumor growth in vivo compared to the empty vector control (Figure S6D), directly demonstrating that MHC‐I is an indispensable downstream hub in the ZBTB21 regulatory pathway. Furthermore, flow cytometric analysis of surface MHC‐I expression (H‐2K/D) under IFN‐γ stimulation or unstimulated conditions provided mechanistic insights: *ZBTB21*‐KO cells showed significantly elevated H‐2Kd/Dd levels; *ZBTB21*‐*B2m*‐KO cells exhibited complete loss of H‐2Kd/Dd expression and unresponsiveness to IFN‐γ; and B2m re‐expression restored both baseline H‐2Kd/Dd expression and IFN‐γ responsiveness (Figure S6E).

Notably, the loss of MHC‐I molecules is widely recognized as a key mechanism of resistance to T cell‐based immunotherapies (e.g., immune checkpoint blockade, ICB) in human cancers [31, 32]. Highly consistent with these clinical observations, treatment of mice bearing 4T1 tumors with blocking anti‐PD‐1 or anti‐CTLA‐4 antibodies effectively induced rejection of *ZBTB21*‐KO tumors and significantly prolonged survival; however, the same treatment was largely ineffective against *ZBTB21*‐*B2m*‐KO tumors (Figure 3H,I; Figure S6F,G). Furthermore, treatment of mice bearing B16F10‐OVA tumors with an mRNA‐lipid nanoparticle (OVA‐LNP) vaccine encoding the OVA antigen (previously shown to induce robust expansion of antigen‐specific cytotoxic CD8^+^ T cells [33, 34]) similarly demonstrated that the OVA‐LNP vaccine significantly suppressed tumor growth and extended survival in the *ZBTB21*‐KO group, while showing no significant effect on *ZBTB21*‐*B2m*‐KO tumor growth (Figure 3J; Figure S6H).

In‐depth analysis of TME changes revealed that flow cytometry of *ZBTB21*‐KO and *ZBTB21*‐*B2m*‐KO tumors showed a significantly reduced proportion of infiltrating CD8^+^ T cells in *ZBTB21*‐*B2m*‐KO tumors (Figure 3K). More critically, key cytokines reflecting T cell activation and function, IFN‐γ and perforin, were significantly diminished (Figure 3L; Figure S6I). These data collectively indicate that MHC‐I deficiency not only reduces CD8^+^ T cell infiltration but, more importantly, impairs their activation and effector function, thereby reversing the antitumor immune gains conferred by ZBTB21 deficiency.

### 2.4 ZBTB21 Coordinately Suppresses GSDMD‐Dependent Pyroptosis and MHC‐I Antigen Presentation Through Epigenetic Mechanisms

**FIGURE 4:**
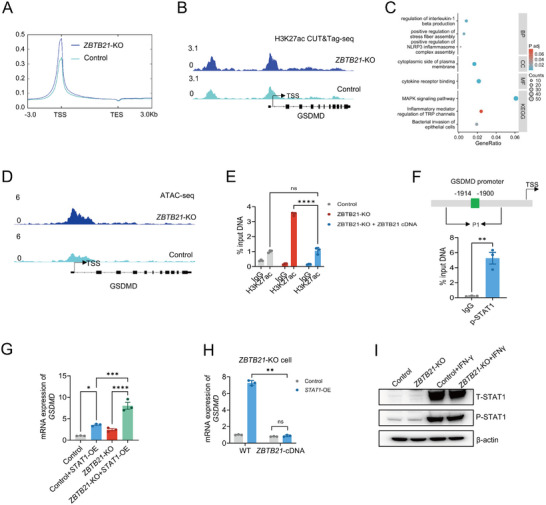
ZBTB21 deficiency upregulates GSDMD via facilitating STAT1‐driven transcription by enhancing chromatin accessibility. (A) Profile around the TSS of H3K27ac‐modified genes. Read counts were extracted for all CUT&Tag‐seq experiments within a region spanning ± 3 kb around the TSS. (B) IGV analysis of H3K27ac peaks at the GSDMD locus with the indicated scale in 4T1 Control and *ZBTB21*‐KO cells. (C) GO and KEGG analyses of genes containing modified regions of H3K27ac. The biological process terms of interest are displayed. The size of a circle indicates the number of enriched genes, and the color reflects the adjusted *p* value (|log2FC| >1 and FDR < 0.05). (D) Distribution of ATAC‐seq signals in the region upstream and downstream of the GSDMD gene transcription start site (TSS). (E) ChIP‐qPCR analysis of H3K27ac antibody‐pulled down chromatins (n = 3). (F) ChIP‐qPCR analysis of p‐STAT1 antibody‐pulled down chromatins (*n* = 3). (G,H) Relative levels of *GSDMD*‐mRNA expression measured by qPCR (*n* = 3). (I) Immunoblotting for STAT1 in 4T1 Control and *ZBTB21*‐KO cells with or without IFN‐γ. Data are presented as mean ± SEM. ^*^ *p* < 0.05; ^**^ *p* < 0.01; ^***^ *p* < 0.001; ^****^ *p* < 0.0001; ns, no significance. Data were analyzed by two‐way ANOVA or unpaired two‐tailed Student's *t*‐test.

To elucidate the mechanism underlying ZBTB21‐mediated GSDMD upregulation, we analyzed ChIP‐seq data and found no significant ZBTB21 binding peaks at the GSDMD transcription start site (TSS) region (Figure S7A), nor was any canonical CRE motif identified within the GSDMD promoter region (consistent with previous reports that ZBTB21 binds CRE sites to suppress target gene expression [26]). This suggested an indirect regulatory mechanism for GSDMD. Considering ZBTB21's reported role in modulating chromatin architecture, we performed genome‐wide H3K27ac profiling using CUT&Tag sequencing. Compared to control cells, ZBTB21 deficient cells exhibited significantly elevated global H3K27ac signals at transcription start site (TSS) regions (Figure 4A), with pronounced enrichment observed at the GSDMD locus (Figure 4B). Functional enrichment analysis of genes associated with increased H3K27ac occupancy in *ZBTB21*‐KO cells revealed significant associations with key biological processes, including interleukin‐1 beta production, NLRP3 inflammasome complex assembly, and MAPK signaling pathways (Figure 4C), validating our previous observations of enhanced pyroptosis upon ZBTB21 depletion. To directly assess the impact of ZBTB21 deficiency on chromatin architecture at the GSDMD locus, ATAC‐seq analysis revealed a marked increase in chromatin accessibility at its promoter region, indicating a physical “opening” of this key genomic locus (Figure 4D). The causal role of ZBTB21 was firmly established by a rescue experiment: re‐expression of ZBTB21 cDNA in *ZBTB21*‐KO cells effectively reversed the enhanced H3K27ac signals at the GSDMD locus, as confirmed by ChIP‐qPCR, restoring them to near wild‐type levels (Figure 4E). Further supporting ZBTB21's role in chromatin organization, its deletion correlated with a looser chromatin conformation (Figure S7B) and a significant reduction in the sequence conservation at H3K27ac‐bound genomic loci (Figure S7C).

Given that STAT1 is a known transcriptional activator of GSDMD [35], we investigated potential ZBTB21‐STAT1 interactions. ChIP‐qPCR assays confirmed robust STAT1 binding to the GSDMD promoter region (Figure 4F). Genetic ablation of ZBTB21 substantially augmented STAT1‐driven *GSDMD* mRNA induction (Figure 4G), an effect that was reversed upon ZBTB21 reconstitution (Figure 4H). Notably, ZBTB21 depletion did not alter STAT1 phosphorylation levels (Figure 4I), suggesting that ZBTB21 modulates STAT1's transcriptional activity primarily through influencing chromatin accessibility, rather than affecting STAT1 activation status.

Having established the epigenetic mechanism for GSDMD regulation, we next investigated how ZBTB21 deficiency enhances MHC‐I expression. Initial analysis of the CUT&Tag data indicated that ZBTB21 deletion did not significantly alter H3K27ac enrichment at the promoters of classical MHC‐I genes H2‐D and H2‐K (Figure S8A,B). Given the complex regulation of MHC‐I expression by numerous transcription factors [36], we systematically analyzed key upstream regulators implicated in MHC‐I transcription. Intriguingly, the H3K27ac levels and mRNA expression of known MHC‐I activators such as *NLRC5* (Figure S8C, D), NF‐κB subunits *p50* and *p65* (Figure S8E–G), and *ATF1* (Figure S8H,I) were largely unaffected by ZBTB21 status.

**FIGURE 5:**
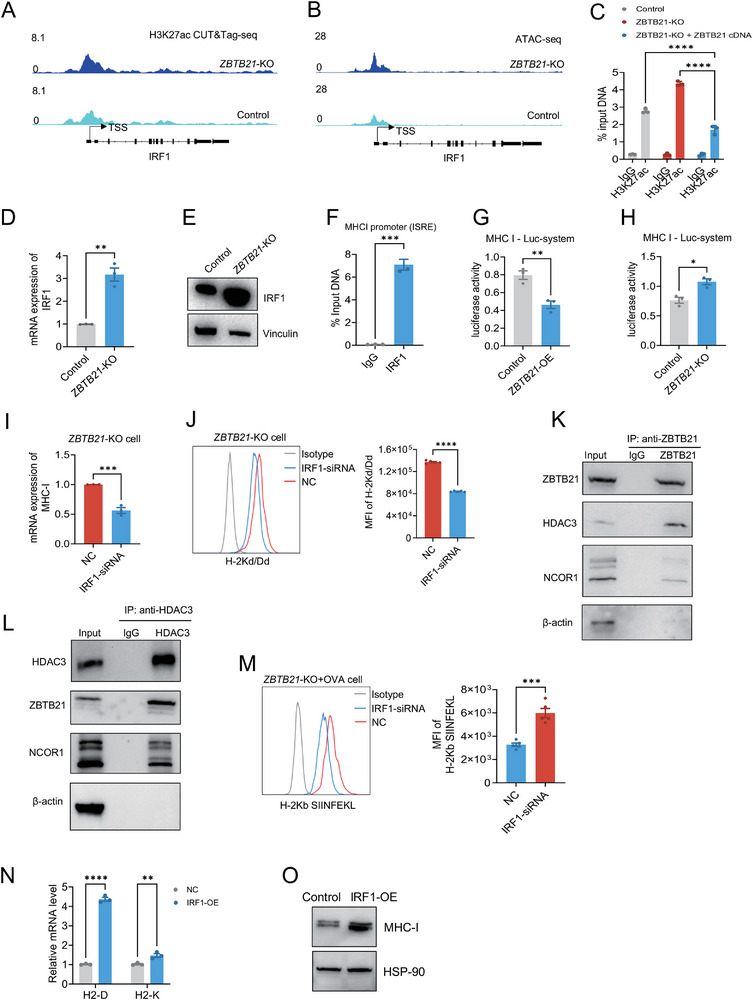
ZBTB21 represses MHC‐I antigen presentation through epigenetic silencing of IRF1. (A) IGV analysis of H3K27ac peaks at the IRF1 locus with the indicated scale in 4T1 Control and *ZBTB21*‐KO cells. (B) Distribution of ATAC‐seq signals in the region upstream and downstream of the IRF1 gene transcription start site (TSS). (C) ChIP‐qPCR analysis of H3K27ac antibody‐pulled down chromatins (*n* = 3). (D) Relative levels of *IRF1*‐mRNA expression measured by qPCR (*n* = 3). (E) Immunoblotting for IRF1 in 4T1 Control and *ZBTB21*‐KO cells. (F) ChIP‐qPCR analysis of IRF1 antibody‐pulled down chromatins. (ISRE, 5'‐GAAANNGAAA‐3'). (G,H) Effect of ZBTB21 overexpression or Knockout on IRF1‐induced activation of the MHC‐I promoter. (I) Relative levels of *MHC‐I*‐mRNA expression measured by qPCR (*n* = 3). (J) Representative staining profiles (left) and H‐2Kd/Dd MFI (right) in *ZBTB21*‐KO cells treated with IRF1‐siRNA or negative control (NC) (*n* = 5). (K,L) Co‐immunoprecipitation analysis of protein complexes associated with ZBTB21. (M) Representative staining profiles (left) and H‐2Kb SIINFEKL MFI (right) in Control+OVA and *ZBTB21*‐KO+OVA cells (*n* = 5). (N) Relative levels of *MHC‐I*‐mRNA expression measured by qPCR (*n* = 3). (O) Immunoblotting for MHC‐I in 4T1 Control and *IRF1*‐KO cells. Data are presented as mean ± SEM. ^*^ *p* < 0.05; ^**^ *p* < 0.01; ^***^ *p* < 0.001; ^****^ *p* < 0.0001. Data were analyzed by unpaired two‐tailed Student's *t*‐test.

Interferon regulatory factor 1 (IRF1) is a critical transcription factor that binds interferon‐stimulated response elements (ISREs) to promote IFNγ‐induced MHC‐I gene expression [37]. IRF1 expression itself is initiated by phosphorylated STAT1 (p‐STAT1) [28]. Importantly, as shown earlier, ZBTB21 deficiency did not alter STAT1 phosphorylation (Figure 4I) or H3K27ac enrichment at the STAT1 promoter (Figure S8J). However, we observed a significant increase in H3K27ac enrichment specifically at the IRF1 TSS in *ZBTB21*‐KO cells compared to controls (Figure 5A). Parallel ATAC‐seq analysis demonstrated that the open chromatin state extended to the IRF1 promoter upon ZBTB21 loss (Figure 5B). Crucially, the increase in H3K27ac at the IRF1 locus was also reversed by ZBTB21 cDNA re‐expression (Figure 5C), demonstrating that ZBTB21 directly constrains the epigenetic state of the IRF1 gene. This epigenetic alteration was functionally consequential, as evidenced by the concomitant upregulation of both IRF1 mRNA and protein levels upon ZBTB21 loss (Figure 5D,E).

To directly evaluate the functional impact of ZBTB21 on IRF1‐mediated MHC‐I activation, ChIP‐qPCR assays confirmed robust IRF1 binding to the MHC‐I promoter ISRE region (Figure 5F). Luciferase reporter assays demonstrated that ZBTB21 overexpression significantly attenuated H‐2K promoter activity in cells transfected with the H‐2K‐luc construct (Figure 5G). Conversely, genetic knockout of ZBTB21 markedly enhanced H‐2K promoter‐driven luciferase activity (Figure 5H). This enhancement was specifically dependent on ZBTB21 loss, as ZBTB21 deficiency significantly potentiated IRF1‐induced upregulation of *MHC‐I* mRNA and protein expression (Figure 5I,J). Thus, ZBTB21 constrains MHC‐I expression by epigenetically restricting IRF1 transcription and functionally dampening IRF1's capacity to activate the MHC‐I promoter. To uncover the core biochemical mechanism underpinning ZBTB21's coordinated epigenetic repression, co‐immunoprecipitation assays verified that ZBTB21 physically interacts with key components of the transcriptional repressor complex, including NCOR1 and HDAC3 (Figure 5K,L). This interaction suggests a model whereby ZBTB21 recruits histone deacetylase complexes to remove activating H3K27ac marks, thereby maintaining a repressive chromatin state at both the Gsdmd and IRF1 loci. These results establish a novel ZBTB21/IRF1/MHC‐I axis critical for tumor immune evasion.

To further validate IRF1 as the pivotal mediator in ZBTB21‐regulated antigen presentation, we knocked down IRF1 in *ZBTB21*‐KO OVA model cells. Flow cytometric analysis revealed that knockdown effectively counteracted the ZBTB21 deficiency‐induced upregulation of SIINFEKL H‐2Kb complex surface expression (Figure 5M). Furthermore, to confirm that IRF1 activation is sufficient to drive MHC‐I upregulation, we overexpressed IRF1 in wild‐type tumor cells. This manipulation significantly enhanced the surface expression levels of MHC‐I molecules (H‐2K/D), demonstrating the potency of IRF1 in activating this critical antigen presentation pathway (Figure 5N,O). In summary, ZBTB21 orchestrates tumor immune evasion by employing distinct epigenetic strategies: it impedes pyroptotic cell death through chromatin remodeling‐mediated attenuation of STAT1‐dependent GSDMD transcription, while concurrently suppressing MHC‐I antigen presentation by restricting both the expression and transactivation capacity of IRF1.

### 2.5 Small‐Molecule Targeting of ZBTB21 Potentiates Antitumor Immunity

**FIGURE 6:**
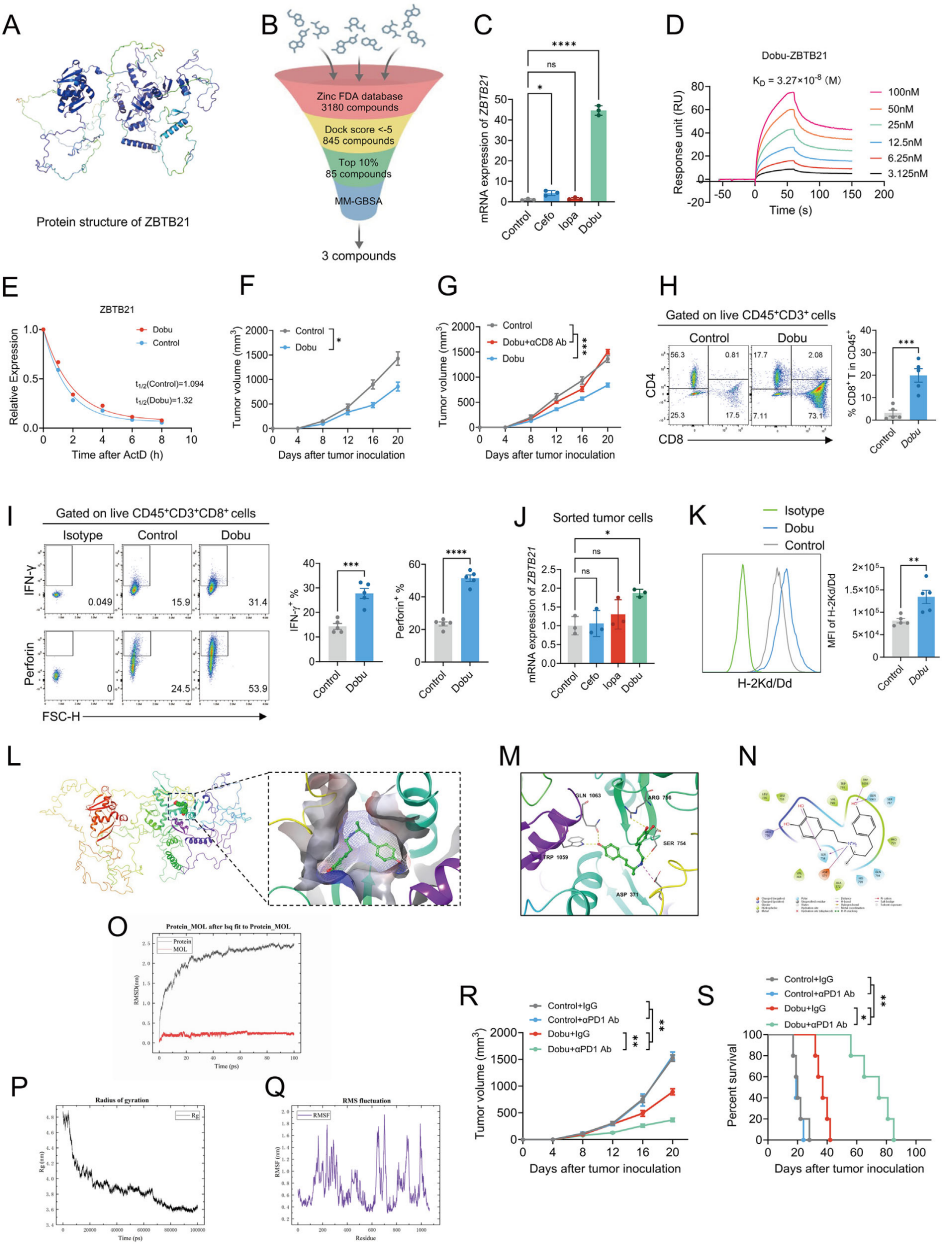
Dobutamine targets ZBTB21 to attenuate transcriptional repression and enhance antitumor immunity. (A) The AlphaFold‐modeled structure of ZBTB21 was refined. (B) Schematic workflow of the docking‐based virtual screening for small‐molecule inhibitors of ZBTB21. (C) Relative levels of *ZBTB21*‐mRNA expression measured by qPCR in 4T1 cells treated with or without Cefoperazone (Cefo), Iopamidol (Iopa), or Dobutamine (Dobu) (*n* = 3). (D) The binding affinity between Dobu and ZBTB21 was determined by surface plasmon resonance. (E) ZBTB21 mRNA decay rate measured by quantitative PCR in cells cultured with Dobu and actinomycin D. (F) Tumor growth curves of 4T1 tumor‐bearing mice treated with or without Dobu (*n* = 6). The results for Figure 6F, Figure S9J,K originated from the same set of experiments. (G) Tumor growth curves of 4T1 tumor‐bearing mice treated with or without Dobu, αCD8, or IgG antibodies (*n* = 6). The results for Figure 6G, Figure S9L,M originated from the same set of experiments. (H) Representative staining profiles (left) of CD4/CD8 and percentages (right) of CD8^+^ T cells among CD45^+^ cells in 4T1 tumor‐bearing mice treated with or without Dobu (*n* = 5). (I) Representative staining profiles (left) and percentages (right) of IFN‐γ and Perforin expression in tumor‐infiltrating CD8^+^ T cells in 4T1 tumor‐bearing mice treated with or without Dobu (*n* = 5). (J) Relative levels of *ZBTB21*‐mRNA expression measured by qPCR in sorted tumor cells from 4T1 tumor‐bearing mice treated with or without Cefo, Iopa, or Dobu (*n* = 3). (K) Representative staining profiles (left) and MFI (right) of H‐2Kd/Dd expression in tumor‐infiltrating CD8^+^ T cells in 4T1 tumor‐bearing mice treated with or without Dobu (*n* = 5). (L–N) Molecular docking and dynamics simulations assessing the binding mode of Dobu with ZBTB21. (O–Q) The stability of the Dobu‐ZBTB21 binding was assessed through root‐mean‐square deviation (N), radius of gyration (O), and root‐mean‐square fluctuation (P). (R,S) Tumor growth and overall survival curves for 4T1 tumor‐bearing mice treated with or without Dobu, αPD1, or IgG antibodies (*n* = 6). Data are presented as mean ± SEM. ^*^ *p* < 0.05; ^**^ *p* < 0.01; ^***^ *p* < 0.001; ^****^ *p* < 0.0001; ns, no significance. Data were analyzed by one‐way ANOVA, two‐way ANOVA, Log‐rank (Mantel‐Cox) test or unpaired two‐tailed Student's *t*‐test.

To translate these mechanistic insights into therapeutic potential, we pursued small‐molecule inhibitors of ZBTB21. Compared to antibodies, small molecules offer superior tissue penetration, enhanced capacity to suppress tumor growth and migration, and favorable biosafety profiles [38, 39]. We first generated the refined protein structure of ZBTB21 (UniProt ID: Q9ULJ3) using AlphaFold [40] (Figure 6A). Virtual screening of FDA‐approved compounds (ZINC database [41]) identified three candidates based on binding free energy: Dobutamine, Cefoperazone, and Iopamidol (Figure 6B; Figure S9A).

In vitro validation revealed that Dobutamine paradoxically increased ZBTB21 levels (Figure 6C; Figure S9B) while simultaneously elevating *MHC‐I* expression (Figure S9C). We hypothesize that Dobutamine inhibits ZBTB21 protein function, triggering a compensatory feedback loop that increases mRNA transcription—consistent with its ability to recapitulate the transcriptional signature of ZBTB21 ablation (MHC‐I upregulation). To provide direct evidence for this interaction, surface plasmon resonance assays confirmed that Dobutamine binds to purified ZBTB21 protein with high affinity (KD = 3.27 × 10^−^^8^ m), demonstrating a specific molecular engagement (Figure 6D). Furthermore, to exclude potential off‐target effects mediated by its known β1 adrenergic receptor (β1‐AR) agonist activity, we pretreated cells with the non‐selective β‐blocker propranolol. Notably, Dobutamine still upregulated MHC‐I expression and induced pyroptosis in the presence of propranolol (Figure S9D,E), indicating that its immunomodulatory effects are independent of β1‐AR signaling.

Functional analyses extended these findings, showing that Dobutamine recapitulates the core phenotypes of ZBTB21 genetic ablation: it enhanced GSDMD‐dependent pyroptosis (Figure S9F), upregulated IRF1 expression (Figure S9G,H), and potentiated the MHC‐I antigen presentation pathway (Figure S9C). Critically, in ZBTB21 knockout tumor‐bearing models, Dobutamine lost its tumor suppressive efficacy, underscoring that its therapeutic actions are strictly dependent on ZBTB21 targeting (Figure S9I). The compensatory increase in *ZBTB21* mRNA upon Dobutamine treatment was further investigated using actinomycin D chase experiments (Figure 6E), which revealed that this upregulation requires de novo transcription, supporting the existence of a feedback loop in response to functional inhibition. In vivo, Dobutamine (Figure 6F)—but not Cefoperazone or Iopamidol (Figures S9J, K)—suppressed tumor growth in orthotopic 4T1 tumor models. Crucially, this effect was strictly CD8^+^ T cell dependent, as CD8^+^ T cell depletion abrogated tumor control (Figure 6G; Figure S9L,M). Despite demonstrating binding to ZBTB21 in silico (Figure S10A,B), Cefoperazone and Iopamidol lacked corresponding antitumor efficacy.

Consistent with functional ZBTB21 inhibition, Dobutamine‐treated tumors exhibited expanded CD8^+^ T cell infiltration (Figure 6H), enhanced IFN‐γ and perforin production (Figure 6I), and upregulated MHC‐I in tumor cells (Figure 6J,K). Molecular docking revealed that Dobutamine occupies the DNA‐binding groove of ZBTB21's C2H2 zinc‐finger domain (Figure 6L), forming a high‐affinity interaction network: a salt bridge with ASP371, hydrophobic stacking with TRP1059, and hydrogen bonds with SER754/GLN1063 (Figure 6M,N). This binding mode directly impairs ZBTB21's DNA‐binding capacity, explaining both the compensatory mRNA increase and functional target derepression. Molecular dynamics simulations confirmed complex stability (RMSD fluctuation < 0.3 nm; Rg≈3.6 nm; Figure 6O,P) and binding‐pocket rigidity (RMSF < 0.1 nm; Figure 6Q). Functionally, Dobutamine synergized with anti‐PD‐1 therapy, enhancing tumor suppression (Figure 6R) and survival (Figure 6S). Thus, Dobutamine exemplifies a pharmacologic strategy to disrupt ZBTB21's protein function despite compensatory mRNA accumulation, thereby co‐activating pyroptosis‐dependent T cell recruitment and MHC‐I‐mediated antigen presentation to overcome ICI resistance.

## 3 Discussion

Although immune ICB has revolutionized cancer therapy, its efficacy remains constrained by tumor‐intrinsic immune evasion mechanisms [1, 2]. This study identifies the transcription factor ZBTB21 as a central epigenetic orchestrator of dual immunosuppressive programs. By simultaneously suppressing GSDMD‐dependent pyroptosis and MHC‐I‐mediated antigen presentation, ZBTB21 establishes an immune‐suppressive microenvironment. Our findings not only elucidate ZBTB21's pivotal role in tumor immune evasion but also provide novel therapeutic targets and combinatorial strategies to overcome ICB resistance (Figure S10C).

Notably, the two pathways coordinated by ZBTB21 are not merely parallel suppression mechanisms but represent a synergistic strategy to cripple distinct phases of the cancer‐immunity cycle. By inhibiting pyroptosis, ZBTB21 prevents the initial “sparking” of immunity—the release of inflammatory signals that recruit and activate innate and adaptive immune cells, thereby maintaining a “cold” tumor state. Concurrently, by repressing MHC‐I expression, it disables the final “execution” phase—the specific recognition and killing of tumor cells by CD8^+^ T cells. This dual blockade ensures that even if immune cells are present, they remain ineffective. Therefore, targeting ZBTB21 simultaneously reactivates both immune priming and effector killing, creating a self‐reinforcing immunogenic cycle that is more potent than targeting either pathway alone.

Mechanistically, ZBTB21 coordinates two synergistic yet distinct pathways: (1) Pyroptosis suppression: Through global modulation of H3K27ac histone modifications, ZBTB21 restricts chromatin accessibility at the GSDMD locus, thereby epigenetically repressing STAT1‐driven GSDMD transcription. This inhibition attenuates inflammasome activation and the release of DAMPs and proinflammatory cytokines, impairing the recruitment and activation of innate and adaptive immune cells. (2) MHC‐I downregulation: By reducing H3K27ac enrichment at the IRF1 promoter, ZBTB21 represses IRF1 transcription and directly impairs IRF1‐mediated transactivation of MHC‐I promoters. This newly identified ZBTB21‐IRF1‐MHC‐I axis results in diminished surface MHC‐I expression and compromised CD8^+^ T cell recognition of tumor antigens. Critically, ZBTB21 executes this dual regulation not through direct promoter binding but by globally remodeling the chromatin landscape to constrain transcription factor activity (STAT1 and IRF1), revealing a non‐canonical epigenetic paradigm distinct from classical transcriptional repressors. Notably, ZBTB21 ablation enhances MHC‐I expression even without exogenous IFNγ stimulation (Figure S5H), demonstrating IFN‐independent regulation of the IRF1‐MHC‐I axis [37] and underscoring ZBTB21's role as a master epigenetic regulator.

Targeting ZBTB21 holds significant potential to overcome ICB resistance. Genetic knockout or pharmacological inhibition of ZBTB21 initiates a self‐reinforcing immunogenic cycle: Pyroptosis‐derived DAMPs and cytokines recruit and activate antigen‐presenting cells and CD8^+^ T cells. Concurrently, upregulated MHC‐I expression optimizes tumor antigen presentation to infiltrating CD8^+^ T cells, enhancing their cytotoxic function (Figure 3D,E). This dual activation drives potent antitumor immunity, significantly suppressing tumor growth and extending survival (Figure 2P–R). The strict dependency on intact MHC‐I antigen presentation was unequivocally demonstrated by B2M‐knockout experiments, which completely reversed therapeutic benefits (Figure 3F,G). Furthermore, ZBTB21 ablation or inhibition synergized with existing immunotherapies—including anti‐PD‐1, anti‐CTLA‐4 antibodies, and therapeutic vaccines—overcoming limitations of monotherapies (Figure 2Q,R; Figure 3H–J). From a translational perspective, our data suggest that ZBTB21 expression could serve as a biomarker to identify patients most likely to benefit from ZBTB21‐targeted therapy. Specifically, tumors characterized by high ZBTB21, low MHC‐I, and a paucity of T cell infiltration—classical “immune‐excluded” or “desert” phenotypes—may be prime candidates for a “ZBTB21 inhibitor + ICB” combination strategy. This approach could potentially convert these immunologically recalcitrant tumors into responsive ones.

Notably, we identified the FDA‐approved drug dobutamine [42, 43] as a potent ZBTB21 inhibitor. Molecular docking and dynamics simulations revealed that dobutamine allosterically inhibits ZBTB21 by occupying its zinc‐finger DNA‐binding groove, forming salt bridges, hydrophobic stacks, and hydrogen bonds (Figure 5L–N). This binding mode disrupts DNA recognition, functionally inactivating ZBTB21 and recapitulating genetic knockout phenotypes: pyroptosis induction (GSDMD cleavage, caspase‐1 activation, IL‐1β/IL‐18 release), MHC‐I upregulation, enhanced CD8^+^ T cell infiltration/function (IFN‐γ, perforin), and synergistic tumor regression with anti‐PD‐1 therapy. Intriguingly, compensatory increases in *ZBTB21* mRNA upon dobutamine treatment did not impede functional suppression, confirming protein‐level inhibition. The superior tissue penetration and low immunogenicity of small‐molecule inhibitors like dobutamine offer clinical advantages over antibody‐based therapies [38, 39]. However, as a known β1‐adrenergic receptor agonist with cardiovascular effects, the repurposing of dobutamine for oncology may be limited by its inherent pharmacology. Our findings therefore, primarily establish the therapeutic principle of pharmacologically targeting ZBTB21. They provide a strong rationale for the future development of novel, highly selective ZBTB21 inhibitors optimized for anticancer use, devoid of adrenergic activity.

Clinically, ZBTB21 expression serves as a biomarker: High levels correlate with poor prognosis, primary anti‐PD‐1 resistance, and reduced CD8^+^ T cell infiltration across cancers, including TNBC. Thus, ZBTB21 expression could stratify patients for “ZBTB21 inhibitor + ICB” combinations, particularly in aggressive tumors where preclinical efficacy was demonstrated (e.g., TNBC, HCC, melanoma).

In summary, we identify ZBTB21 as a druggable epigenetic nexus coordinating pyroptosis resistance and antigen presentation escape. Pharmacological disruption of ZBTB21 co‐activates the priming phase (pyroptosis‐mediated immune cell recruitment) and effector phase (MHC‐I‐dependent tumor cell killing), overcoming two major barriers to ICB efficacy: inadequate T cell infiltration in “cold” tumors and dysfunctional cytotoxicity due to MHC‐I defects. This work provides a unifying combinatorial strategy to reinvigorate antitumor immunity by synchronously targeting immune priming and effector execution.

## 4 Experimental Section/Methods

### 4.1 Animals

C57BL/6 and Balb/c mice were purchased from Charles River (Beijing, China); NCG (NOD/ShiLtJGpt‐Prkdcem26Cd52Il2rgem26Cd22/Gpt) mice were purchased from Shanghai Model Organisms (Shanghai, China). OT‐I mice were purchased from GemPharmatech (Nanjing, China). All experimental animals were kept and operated in accordance with the guidelines of the Animal Experiment Ethics Committee of The First Affiliated Hospital of Shandong First Medical University & Shandong Provincial Qianfoshan Hospital (Approval No. 2024060401).

### 4.2 Cell Lines

Mouse triple‐negative breast cancer cell line 4T1 (Cat# CRL‐2539), mouse melanoma cell line B16F10 (Cat# CRL‐6475), mouse hepatocellular carcinoma cell line Hepa1‐6 (Cat# CRL‐1830), human triple‐negative breast cancer cell line MDA‐MB‐231 (Cat# CRM‐HTB‐26), human malignant melanoma cell line A375 (Cat# CRL‐1619), human non‐small cell lung cancer cell line H1299 (Cat# CRL‐5803), and human embryonic kidney 293T (Cat# CRL‐3216) cells were obtained from the American Type Culture Collection. All cell lines were maintained in DMEM (Gibco, Cat#C11995500BT) or 1640 (Gibco, Cat#C11875500BT) supplemented with 10% fetal bovine serum (Transgen, Cat#FS301‐02), 100 U/mL penicillin, and 100 µg/mL streptomycin (Transgen, Cat#FG101‐01). Cells were cultured in a humidified incubator at 37°C with 5% CO2. All cell lines have been confirmed to be free of contamination.

### 4.3 CRISPR/Cas9 Knockout Cell Lines

Two single‐guide RNAs (sgRNAs) were designed for each target gene to generate corresponding knockout cell lines. The sgRNAs were designed using the Optimized CRISPR Design tool (https://zlab.bio/guide‐design‐resources), with specific sequences listed in Table S1. The pL‐CRISPR.EFS.tGFP plasmid (Addgene, Cat#57818) was digested with FastDigest Esp3I (Thermo Fisher Scientific, Cat#FD0454) following the manufacturer's instructions. Annealed sgRNA oligonucleotides were ligated into the digested plasmid using T4 DNA Ligase (Thermo Fisher Scientific, Cat#EL0011). The ligation products were transformed into Stbl3 competent cells (TransGen Biotech, Cat#CD521‐02) and cultured on ampicillin‐resistant plates. After colony formation, selected colonies were grown in liquid culture, and plasmids were extracted using the Plasmid Mini Kit (Tiangen, Cat#DP103‐02). Successful ligation was confirmed by PCR and Sanger sequencing. The verified plasmids were then transiently transfected into target cells using Lipofectamine 2000 (Invitrogen, Cat#11668019). After 24 h of culture, GFP fluorescence was observed under a fluorescence microscope (Olympus, Model IX71) to confirm transfection efficiency. Transfected cells were sorted using a BD Influx cell sorter (BD Biosciences) to establish monoclonal cell lines in 96‐well plates for subsequent experiments.

### 4.4 Gene Identification of Knockout Clones

Following colony formation, monoclonal cells were transferred to a 24‐well plate and cultured until reaching 80%–90% confluence. Genomic DNA was extracted using the Genomic DNA Extraction Kit (Bimake, Cat#B40015) according to the manufacturer's instructions. The extracted DNA was amplified by PCR using primers listed in Table 1, and the resulting amplicons were subjected to sequencing. Sequence alterations were analyzed using SnapGene software (GSL Biotech LLC). Clones exhibiting deletions or insertions of non‐triplet base numbers in both chromosomes were selected for further validation by Western blot analysis.

### 4.5 Construction of Overexpression Cell Lines Using the Lentiviral System

Stable overexpression cell lines were generated using a third‐generation lentiviral packaging system. The target gene was cloned into the pCDH‐CMV‐MCS‐EF1‐CopGFP‐T2A‐Puro vector (NovoPro, Cat#V012884). HEK293T cells were seeded at 70% confluence in 10 cm dishes and co‐transfected with the recombinant pLenti vector, packaging plasmid psPAX2 (Addgene, Cat#12260), and envelope plasmid pMD2.G (Addgene, Cat#12259) at a molar ratio of 4:3:1 using Lipofectamine 2000 (Invitrogen, Cat#11668019). At 48 and 72 h post‐transfection, viral supernatants were collected, filtered through a 0.45 µm filter, and concentrated using ultracentrifugation at 3500 rpm for 40 min at 4°C. Target cells were seeded at 50% confluence and infected with the concentrated lentivirus in the presence of 8 µg/mL polybrene (Hanheng Biotechnology, Cat#HB‐PB‐500). After 24 h, the medium was replaced with fresh culture medium. At 48 h post‐infection, cells were selected with 2 µg/mL puromycin (Gibco, Cat#A1113803) for 7 days to establish stable overexpression cell lines. Overexpression was confirmed by Western blot analysis.

### 4.6 Cell Pyroptosis Induction

4T1 and *ZBTB21*‐KO cells were collected and seeded into a 48‐well plate at a density of 8 × 10^4^ cells per well. After cell adhesion, Lipopolysaccharides (LPS, Sigma, Cat#L2654) were added at a concentration of 1 µg/ml and incubated overnight. Subsequently, Nigericin (MCE, Cat#HY‐127019) was introduced at a concentration of 20 µm, and the cultures were maintained for an additional 24–48 h to induce pyroptosis. Pyroptosis was confirmed by propidium iodide (PI) staining to assess plasma membrane integrity, along with observation of characteristic morphological features such as cellular swelling and dynamic bubble‐like protrusions. The aggregation of the pyroptosis execution protein GSDMD‐N on the plasma membrane was detected using anti‐GSDMD‐N immunofluorescence. Finally, both cells and culture supernatant were collected for subsequent analyses.

### 4.7 Sorting of CD4^+^ and CD8^+^ T Cells

Single‐cell suspensions were prepared from mouse spleen or tumor tissues. Cells were stained with PE‐anti‐CD4 (Biolegend, Cat#130310) and PE/CY5‐anti‐CD8 (Biolegend, Cat#100710) antibodies at 4°C for 30 min in the dark. After washing twice with phosphate‐buffered saline (PBS), cells were incubated with anti‐PE magnetic beads (Miltenyi Biotec, Cat#130‐048‐801) at 4°C for 30 min. Following two additional washes, the cell suspension was passed through an LS magnetic separation column (Miltenyi Biotec, Cat#130‐042‐401) to positively select CD4^+^ and CD8^+^ T cells.

### 4.8 Western blot Analysis

A total of 1 × 10^6^ cells were collected and pelleted in 1.5 mL EP tubes. For whole‐cell protein extraction, cells were resuspended in 100 µL RIPA lysis buffer (Invitrogen, Cat#P0013E) and lysed at 4°C for 15 min. The lysates were centrifuged at 12,000 rpm for 10 min, and the supernatants were transferred to new EP tubes. For nuclear protein extraction, cells were processed using the Nuclear Protein Extraction Kit (Keji Biotech, Cat#KGBSP002). Briefly, cells were harvested, washed with PBS, and lysed with cytoplasmic extraction buffer to isolate the cytoplasmic fraction. The nuclear pellet was resuspended and lysed in nuclear extraction buffer to obtain nuclear protein extracts. Protein concentrations of both whole‐cell and nuclear extracts were determined using a BCA assay (Thermo Scientific, Cat#23227). Samples were then mixed with loading buffer, denatured by boiling at 100°C for 10 min, and stored at ‐20°C for subsequent analyses. 50 ug total protein per sample was loaded for sodium dodecyl sulfate‐polyacrylamide gel electrophoresis analysis. Protein samples were loaded onto 4%–12% SDS‐PAGE precast gels (Smart‐Lifesciences, Cat#SLE019) for electrophoresis, and then the gels were blotted to nitrocellulose membranes. Nitrocellulose membranes were blocked with 5% skim milk in Tris‐buffered saline with Tween‐20 (TBST, 10 mm Tris pH 8.0, 150 mm NaCl, and 0.1% Tween 20) for 1 h at room‐temperature. Membranes were then incubated overnight with the primary antibody (1:1000 dilution) in 5% bovine serum albumin (BSA)‐TBST buffer. Membranes were washed and incubated with Anti‐rabbit/mouse IgG, HRP‐linked Antibody (1:5000 dilution) for 1 h at room‐temperature. After washing, the membrane was visualized with High‐sig ECL Western Blotting Substrate (Tanon, Cat#180‐5001). Western‐blot antibodies: Anti‐GSDMD n‐ternal (Biodragon, Cat#BD‐PT7991), Anti‐Caspase1 (Cell signal technology, Cat#83383), Anti‐ZBTB21 (Mybiosource, Cat#MBS9602876), Anti‐STAT1(Cell signal technology, Cat#9172), Anti‐p‐STAT1 (Abcam, Cat#ab109461), Anti‐Zap70 (Selleck, Cat#F0458), Anti‐p‐Zap70 (Selleck, Cat#F0556), Anti‐PI3K(Selleck, Cat#F0294), Anti‐p‐PI3K (Abcam, Cat#ab182651), Anti‐JNK (Cell signal technology, Cat#9252T), Anti‐p‐JNK (Cell signal technology, Cat#9255S), Anti‐LCK (Abcam, Cat#ab227975), Anti‐p‐LCK (Abcam, Cat#ab318960), Anti‐ERK1/2 (Cell signal technology, Cat#4695T), Anti‐p‐ERK1/2 (Cell signal technology, Cat#4695T), Anti‐P38 (Cell signal technology, Cat#8690T), Anti‐p‐P38 (Cell signal technology, Cat#4511T), Anti‐Histone H3 (Cell signal technology, Cat#4658S), Anti‐IRF‐1 (Cell signal technology, Cat#8478), Anti‐Vinculin (Cell signal technology, Cat#4650S), Anti‐β‐actin (Cell signal technology, Cat#3700), Anti‐rabbit IgG HRP‐linked Antibody (Cell signal technology, Cat#7074), Anti‐mouse IgG HRP‐linked Antibody (Cell signal technology, Cat#7076), Anti‐HDAC3 Recombinant Rabbit Monoclonal Antibody (HUABIO, Cat#HA750221), Anti‐Nuclear Receptor Corepressor Mouse Monoclonal Antibody (HUABIO, Cat#HA600055), Anti‐HLA‐A/B/C (MHC I) Recombinant Rabbit Monoclonal Antibody (HUABIO, Cat#HA723415), Anti‐IRF1 Recombinant Rabbit Monoclonal Antibody (HUABIO, Cat#ET1602‐28), and Anti‐ZNF295 Polyclonal Antibody (Invitrogen,Cat#PA5‐99758).

### 4.9 Tumor Models and Treatments

For 4T1 tumor models, 4T1 cells (including knockout and overexpression cell lines) were harvested and resuspended in PBS at a concentration of 2 × 10^6^ cells/mL. Following anesthesia, a small incision was made in the skin beneath the upper right nipple of female Balb/c mice, and 2 × 10^5^ cells (100 µL) were injected into the subcutaneous breast tissue in situ. For B16F10 and Hepa1‐6 tumor models, cells were collected and adjusted to concentrations of 5 × 10^6^ and 2 × 10^7^ cells/mL in PBS, respectively. After anesthetizing C57BL/6 mice, the fur on the right dorsal region was shaved, and 5 × 10^5^ B16F10 cells or 2 × 10^6^ Hepa1‐6 cells (100 µL) were subcutaneously inoculated into the right dorsal area. The tumor size was monitored every day, and the long (a) and short (b) diameters of the tumor were measured with a vernier caliper every 4 days. The volume of the tumor was calculated as tumor volume = ab^2^/2. Mice were euthanized at the indicated time points or when the tumor volume reached 2000 mm^3^. For survival studies, survival time was recorded as the duration from the date of tumor implantation until natural death or euthanasia. To deplete CD8^+^ T cells and CD19^+^ B cells, mice were intraperitoneally injected with 0.25 mg of InVivoMAb anti‐mouse CD8α antibody (Bioxcell, clone 53–6.7, Cat#BE0117), InVivoMAb anti‐mouse CTLA‐4 antibody (Bioxcell, clone UC10‐4F10‐11, Cat#BE0032), InVivoMAb anti‐mouse CD19 antibody (Bioxcell, clone 1D3, Cat#BE0150), InVivoMAb rat IgG2b antibody (Bioxcell, clone LTF‐2, Cat#BE0090), or InVivoMAb rat IgG2a antibody (Bioxcell, clone 2A3, Cat#BE0089) on days 6 and 12 following tumor inoculation. For macrophage depletion, 200 µL of Clodronate liposomes (Liposoma, Cat#C‐005) was administered intraperitoneally on days 8, 12, and 16 post‐inoculation. To deplete NK cells, 30 µL of anti‐asialo GM1 antibody (Wako Chemicals, Cat#014‐09801) was injected intraperitoneally every other day. The efficiency of depletion was confirmed by flow cytometry analysis of CD8^+^ T cells, CD19^+^ B cells, macrophages, and NK cells in the spleen, using established protocols. OVA mRNA‐LNP was purchased from Hepsin Biotechnology (Shanghai) Co., Ltd. For tumor‐bearing mouse immunization, 40 µg of OVA mRNA‐LNP or empty vector control mRNA‐LNP was administered via retro‐orbital (r.o.) injection on days 5, 8, 12, and 19 after tumor inoculation. For in vivo treatment, every tumor‐bearing mouse was intraperitoneally injected with 100 µg Dobutamine (MCE, Cat#HY‐15746), 200 µg Iopamidol (MCE, Cat#HY‐B0684), or 2 mg Cefoperazone (MCE, Cat#HY‐B0210) every other day, starting from the day of tumor inoculation. InVivoMAb anti‐mouse PD‐1 antibody (Bioxcell, clone 29F.1A12, Cat#BE0273) or InVivoMAb rat IgG2a antibody (Bioxcell, clone 2A3, Cat#BE0089) was administered intraperitoneally at a dose of 200 µg on days 6 and 12 post‐tumor inoculation.

### 4.10 Transcriptome Sequencing and Data Analysis

Total RNA was extracted from 4T1 and *ZBTB21*‐KO cells collected in 1.5 mL EP tubes following digestion and centrifugation. Each sample was lysed in 1 mL TRIzol Reagent (Invitrogen, Cat#15596026CN), and RNA was isolated according to the manufacturer's protocol. Sequencing libraries were constructed using the NEBNext Ultra RNA Library Prep Kit for Illumina (NEB, USA) as per the manufacturer's instructions, with index codes added to assign sequences to individual samples. Library clustering was performed on a cBot Cluster Generation System using the TruSeq PE Cluster Kit v3‐cBot‐HS (Illumina) following the manufacturer's guidelines. The prepared libraries were sequenced on an Illumina HiSeq platform, generating 125 bp or 150 bp paired‐end reads. Raw FASTQ data were processed using in‐house Perl scripts to obtain clean reads by removing adapter‐containing reads, poly‐N sequences, and low‐quality reads. The reference genome index was built with Hisat2 (v2.0.5), and paired‐end clean reads were aligned to the reference genome using Hisat2. Read counts per gene were quantified using featureCounts (v1.5.0‐p3). Gene expression levels were calculated as Fragments Per Kilobase of transcript per Million mapped reads (FPKM) based on gene length and mapped read counts. Read counts were normalized using the edgeR package with a scaling factor.

### 4.11 Flow Cytometry

For flow cytometry analysis of tumor tissue, the in situ tumor was excised after carefully removing the surrounding skin. The tumor was placed in a 4 mL EP tube with 2 mL DMEM, minced into 1 mm^3^ fragments, and transferred to a 50 mL centrifuge tube. Following centrifugation at 1800 rpm for 5 min, the supernatant was discarded. The tissue fragments were resuspended in 10 mL of Hank's Solution containing 2 mg/mL collagenase I (Gibco, Cat#17100017) and digested at 37°C with shaking at 140 rpm for 50 min. Digestion was terminated by adding an equal volume of complete medium, and the resulting cell suspension was filtered through a 40 µm cell strainer into a new 50 mL centrifuge tube. After centrifugation at 1800 rpm for 5 min, the supernatant was removed, and the pellet was resuspended in 1 mL of erythrocyte lysis buffer (Solarbio, Cat#R1010) for 10 min on ice to lyse red blood cells. The suspension was centrifuged again at 1800 rpm for 5 min, the supernatant was discarded, and the pellet was resuspended in an appropriate volume of FACS buffer to obtain a single‐cell tumor suspension. For spleen sample preparation, the spleen was removed, placed in a 6 cm dish, and mechanically dissociated into a cell suspension. The suspension was collected into a 15 mL centrifuge tube, centrifuged at 1800 rpm for 5 min, and the supernatant was discarded. Red blood cells were lysed using 1 mL of erythrocyte lysis buffer (Solarbio, Cat#R1010) for 10 min on ice. After centrifugation at 1800 rpm for 5 min, the supernatant was removed, and the pellet was resuspended in FACS buffer to yield a single‐cell suspension.

A total of 1 × 10^6^ cells were transferred to a flow cytometry tube and blocked with 0.25 µL of anti‐CD16/32 for 10 min at room‐temperature to prevent non‐specific binding. Cells were then incubated with fluorochrome‐conjugated antibodies for 30 min on ice in the dark, washed twice with FACS buffer, and resuspended in 200 µL of FACS buffer. For intracellular cytokine staining, cells were stimulated for 5 h with Leukocyte Activation Cocktail (BD Biosciences, Cat#550583). Subsequently, cells were stained for surface antigens, washed twice with FACS buffer, and fixed and permeabilized using Cytofix/Cytoperm solution (BD Biosciences, Cat#555028). Intracellular staining was performed with BV785‐anti‐IFNγ and PE‐anti‐Perforin. Cells were washed twice with wash buffer, centrifuged, and resuspended in 200 µL of FACS buffer. All samples were acquired using a Cytek Aurora flow cytometer (Cytek Biosciences, Fremont, CA), and data were analyzed using FlowJo software (Tree Star). Fluorescent antibodies: Anti‐mouse CD16/32 (Biolegend, Cat#101320), BV570‐anti‐CD45 (Biolegend, clone 30‐F11, Cat#103136), PE/Dazzle594‐anti‐CD3 (Biolegend, clone 17A2, Cat#100246), PE‐anti‐CD4 (Biolegend, clone GK1.5, Cat#130310), PE/CY5‐anti‐CD8a (Biolegend, clone 53–6.7, Cat#100710), AF700‐anti‐CD19 (Biolegend, clone 6D5, Cat#115528), BV605‐anti‐CD103 (Biolegend, clone 2E7, Cat#121433), BV605‐anti‐CD11b (Biolegend, clone M1/70, Cat#101237), PERCP/CY5.5‐anti‐Gr‐1 (Biolegend, clone RB6‐8C5, Cat#108428), AF647‐anti‐F4/80 (Biolegend, clone BM8, Cat#123122), PE/CY7‐anti‐CD206 (Biolegend, clone C068C2, Cat#141720), FITC‐anti‐CD28 (Biolegend, clone E18, Cat#122007), PE‐anti‐NKp46 (Biolegend, clone 29A1.4, Cat#137604), APC/CY7‐anti‐NKp46 (Biolegend, clone 29A1.4, Cat#137646), Pacific Blue‐anti‐CD49b (Biolegend, clone DX5, Cat#108918), PERCP/CY5.5‐anti‐CD31 (Biolegend, clone MEC13.3, Cat#102522), APC‐anti‐CD90.2 (Biolegend, clone 30‐H12, Cat#105312), PE/CY7‐anti‐PD‐L1 (Biolegend, clone 10F.9G2, Cat#124314), BV421‐anti‐PD‐1 (Biolegend, clone 29F.1A12, Cat#135221), APC‐anti‐TIM3(Biolegend, clone B8.2C12, Cat#134008), BV785‐anti‐IFN‐γ (Biolegend, clone XMG1.2, Cat#505838), PE‐anti‐Perforin (Biolegend, clone S16009A, Cat#154306), APC anti‐mouse H‐2Kb bound to SIINFEKL Antibody (Biolegend, clone 25‐D1.16, Cat#141606), PerCP/Cyanine5.5 anti‐mouse H‐2Kd/H‐2Dd Antibody (Biolegend, clone 34‐1‐2S, Cat# 114716) and APC anti‐mouse H‐2Kd Antibody (Biolegend, clone SF1‐1.1, Cat#116620).

### 4.12 Quantitative Real‐Time PCR

Cell RNA was extracted with TRIzol (Thermo Fisher, Cat#15596026CN), and the RNA concentration was detected by a NanoDrop spectrophotometer (Thermo Fisher). 1 µg of RNA was then reverse transcribed to cDNA using HiScript II Q RT SuperMix (Novizan, Cat#R223‐01). 1 µL of cDNA was used for the next step of quantitative real‐time PCR (qPCR) with ChamQ Universal SYBR qPCR Master Mix (Novizan, Cat#Q711‐03). PCR procedures were performed on the Applied Biosystems StepOnePlus Real‐Time PCR System Thermal Cycling Block. The mRNA level of target genes was calculated with the 2^^−ΔΔCt^ method, and data were normalized to the expression of β‐actin as the housekeeping gene. The primers used for the qPCR reactions are shown in Table S1.

### 4.13 Elisa

Mouse serum and cell culture supernatant levels of IL‐1β and IL‐18 were quantified using the Mouse IL‐1β ELISA Kit (Shanghai Enzyme‐linked Biotechnology, Cat#ML098416) and Mouse IL‐18 ELISA Kit (Shanghai Enzyme‐linked Biotechnology, Cat#ML002294), respectively, according to the manufacturer's instructions. Serum was obtained by cardiac puncture, clotted for 30 min, and centrifuged at 3000 rpm for 10 min at 4°C. Cell culture supernatants were centrifuged at 1000 rpm for 5 min to remove debris. Standards and samples (100 µL) were added to pre‐coated 96‐well plates in duplicate, incubated at 37°C for 90 min, and washed five times with wash buffer. Biotinylated detection antibodies were added, incubated at 37°C for 60 min, followed by HRP‐streptavidin for 30 min at 37°C. After washing, TMB substrate was added, incubated for 15–20 min in the dark, and stopped with the stop solution. Absorbance was measured at 450 nm using a microplate reader (TECAN, Spark).

### 4.14 Cell Proliferation

Cell viability was assessed using the Enhanced Cell Counting Kit‐8 (Beyotime, Cat#C0042) according to the manufacturer's instructions. Briefly, cells were seeded in 96‐well plates at a density of 5 × 10^3^ cells per well in 100 µL of complete culture medium and incubated at 37°C in a 5% CO_2_. Cell viability was measured at multiple time points (0, 24, 48, and 72 h). At each time point, 10 µL of Enhanced CCK‐8 reagent was added to each well, followed by incubation for 1–2 h at 37°C in the dark. Absorbance was recorded at 450 nm using a microplate reader (TECAN, Spark). Each experimental group was set up in triplicate wells to ensure reproducibility.

### 4.15 Cell Migration—Scratch Assay

Cells were seeded in six‐well plates at a density of 5 × 10^5^ cells per well in 2 mL of complete culture medium and incubated at 37°C in a 5% CO_2_ atmosphere until reaching 90%–100% confluence. A sterile 200 µL pipette tip was used to create a uniform scratch across the cell monolayer in each well, and the wells were gently washed twice with PBS to remove detached cells and debris. Add fresh serum‐free medium and continue incubation. Images of the scratch were captured at 0, 12, and 24 h post‐scratch using a microscope (Olympus, Model IX71) equipped with a digital camera at 10× magnification. The scratch width was measured at multiple points along the scratch using ImageJ software (NIH, Bethesda, MD). The migration rate was calculated as the percentage of scratch closure, determined by the formula: [(initial scratch width at 0 h—scratch width at time t) / initial scratch width] × 100%. Each experimental group was set up in triplicate wells to ensure reproducibility.

### 4.16 CUT&Tag Assay

The CUT&Tag assay was performed on 1 × 10^6^ 4T1 or *ZBTB21*‐KO cells using the Hyperactive Universal CUT&Tag Assay Kit for Illumina (Vazyme), following the manufacturer's instructions. Briefly, cells were permeabilized with concanavalin A‐coated magnetic beads and digitonin to enhance membrane accessibility. Subsequently, cells were incubated overnight at 4°C with primary antibodies: either anti‐H3K27ac (1:500; Cell Signaling Technology, Cat#8173) or anti‐HA (1:100; Santa Cruz Biotechnology, Cat#sc‐7392 X). Through sequential binding of the primary antibody to the target protein and the secondary antibody to Protein A/G, Tn5 transposase fused to Protein A/G was precisely guided to cleave DNA adjacent to the target protein‐binding sites. During cleavage, the transposase simultaneously ligated adapter sequences to both ends of the DNA fragments. These adapter‐ligated fragments were then amplified by PCR using the TD202 TruePrep Index Kit V2 for Illumina (Vazyme) to construct sequencing libraries. Libraries were sequenced on the Illumina NovaSeq 6000 platform. Peak calling was performed via sparse enrichment analysis, and subsequent data analysis utilized the HOMER software suite for CUT&Tag‐specific processing.

### 4.17 Cytotoxicity Assay

To evaluate the cytotoxicity of CD8^+^ T cells, we performed a lactate dehydrogenase (LDH) cytotoxicity assay (Promega, Cat. No. G1780) according to the manufacturer's instructions. After co‐culturing Control+OVA or *ZBTB21*‐KO+OVA B16F10 cells with OT‐I CD8^+^ T cells, 50 µL of the co‐culture supernatant was collected and transferred to a 96‐well plate. An equal volume of CytoTox 96 Reagent was then added, followed by incubation at room‐temperature for 30 min. Subsequently, the absorbance was measured at 490 nm using a full‐wavelength microplate reader (TECAN, Infinite M200 PRO) to determine the level of cytotoxicity.

### 4.18 Co‐Culture

Spleens from OT‐I mice were processed through a 40 µm cell strainer to generate single‐cell suspensions, followed by red blood cell lysis to isolate splenocytes. The splenocytes were then counted. CD8^+^ T cells were isolated from OT‐I mouse splenocytes using a CD8^+^ T Cell Isolation Kit (Miltenyi Biotec, Cat. No. 130‐096‐543). The isolated CD8^+^ T cells were cultured in RPMI 1640 medium supplemented with 10% fetal calf serum (FCS), 2 µg/mL OVA peptide (Sigma, Cat. No. S7951), 100 µmol/L β‐mercaptoethanol, and 5 ng/mL murine interleukin‐2 for 2 days to induce T cell activation. The activated CD8^+^ T cells were then seeded into a 12‐well plate and co‐cultured with B16F10‐OVA cells at a ratio of 1:5 (tumor cells to immune cells). CD8^+^ T cells grew in suspension, while B16F10‐OVA cells were adherent. After co‐culture, suspended cells were collected for flow cytometry analysis. This study was conducted in accordance with the principles of the Helsinki Declaration (2013 revision) and was approved by the Ethics Committee of The First Affiliated Hospital of Shandong First Medical University & Shandong Provincial Qianfoshan Hospital (Approval No. 2025‐S879). Written informed consent was obtained from each participant prior to the experimental procedures. Peripheral blood was collected from four healthy donors using EDTA anticoagulant tubes. Human T cells were isolated immediately after collection. Primary human T cells were enriched from buffy coats using the RosetteSep Human T Cell Enrichment Cocktail (STEMCELL Technologies) according to the manufacturer's instructions. *ZBTB21*‐KO MDA‐MB‐231 cells were seeded into a 12‐well plate and co‐cultured with activated human T cells at a ratio of 1:10 (tumor cells to immune cells). Human T cells were activated by stimulation with 2 µg/mL anti‐human CD3 antibody and anti‐human CD28 antibody. After co‐culture, cells were analyzed by flow cytometry.

### 4.19 Immunohistochemistry (IHC)

For IHC staining, a lung adenocarcinoma tissue microarray (array number: HLugA030PG04‐1; batch number: XT22‐001‐2) purchased from Shanghai Superchip Biotech Co., Ltd. was used. The ethical approval number for this project is SHYJS‐CP‐2206003. Tissue sections were stained with an anti‐human MHC‐I antibody (Cat#HA723415, HUABIO) or an anti‐human ZBTB21 antibody (Cat#PA5‐99758, Invitrogen). Immunoreactivity was detected using the UltraSensitive Streptavidin‐Peroxidase kit (Product Code: KIT‐9706, Myxin) according to the manufacturer's instructions.

### 4.20 Chromatin Immunoprecipitation (ChIP) Assay

Chromatin was extracted from MDA‐MB‐231 cells after fixation with formaldehyde. Anti‐IRF‐1 (Cell signal technology, Cat#8478), Anti‐H3K27ac (Invitrogen, Cat#MA5‐42798), Anti‐p‐STAT1 (Abcam, Cat#ab109461) and anti‐IgG (Cell Signaling Technology, Cat#2729) were used for the immunoprecipitation of chromatin with an SimpleChIP Plus Enzymatic Chromatin IP Kit (Cell Signaling Technology, Cat#9005s) according to the manufacturer's instructions. The input DNA and immunoprecipitated DNA were purified and detected by qPCR.

### 4.21 Dual Luciferase Reporter Assay

The full‐length promoters of MHC‐I were respectively cloned into luciferase reporter vectors. 4T1, *ZBTB21*‐OE or *ZBTB21*‐KO 4T1 cells were co‐transfected with these reporter vectors. After 48 h, cells were harvested, and the activities of firefly and Renilla luciferase were quantified using the Duo‐Lite Luciferase Assay System (Vazyme, Cat# DD1205‐01).

### 4.22 Alphafold to Predict the Crystal Structure of the Protein

The multimer model in Alphafold v2.3.1 was used to predict the multimeric structure of the protein. Pymol v2.5.0 was used to analyze the predicted multimer structures to obtain the amino acid pairs that can interact to form hydrogen bonds between proteins. The predicted multimer structures were analyzed using PRODIGY v2.0 to get the multimers' binding affinity and dissociation constants formed between different proteins.

### 4.23 Virtual Screening Workflow

Receptor and ligand preparation. The structure of Q9ULJ3 was obtained via AlphaFold modeling, and the ZBTB21 protein structure was optimized. The Protein Preparation Wizard panel in Schrödinger (2021) was used to add hydrogen atoms, assign charges and protonation states at pH 7.0, and minimize the structure using the OPLS‐2005 force field. In Schrödinger, the preprocessed structure was used to generate a grid with the Glide 6.6 module. The helical structure was defined as the center, and the grid box was set to 30 × 30 ×3 0 Å. The FDA dataset from the ZINC database was used as the screening source. All compounds were imported into the Ligprep 3.3 module in Schrödinger for ligand preparation, which involved generating ionization states at pH 7.0 ± 2.0 using Epik 3.1, creating stereoisomers, and producing one low‐energy conformation per ligand. Docking‐based virtual screening. The generated grid and prepared ligands were subjected to virtual screening using Glide 6.6 in three tiers: 1. Glide‐SP for high‐throughput screening: Molecules with Glide scores < ‐5 were retained for the next step. 2. Glide‐XP for fine screening: The top 10% of molecules were selected. 3. Prime MM‐GBSA binding energy calculation: The binding free energies of the final 85 molecules with Q9ULJ3 were computed using the Prime MM‐GBSA method in Maestro.

### 4.24 Detecting mRNA Stability

*ZBTB21* mRNA stability was detected according to the experimental method reported by Hamilton et al. [44]. Briefly, 3 × 10^5^ 4T1 cells were cultured in a six‐well plate for 6 h. Cells were cultured for 24 h after treated with Dobu. Following the addition of 10 µg/mL actinomycin D, cells were harvested at 0, 1, 2, 4, 6 and 8 h for total RNA extraction. The half‐life of *ZBTB21* mRNA was sensitively determined by quantitative PCR. The average Ct value of each time point is normalized to the average Ct value of t = 0, and the value ∆Ct is obtained. ∆Ct = (Average Ct of each time point – Average Ct of t = 0). The relative abundance of each time point is calculated. mRNA abundance = 2^−∆CT^. The relative abundance of mRNA relative to t = 0 at each time point was plotted using GraphPad Prism software.

### 4.25 SPR (surface plasmon resonance)

SPR assay of recombinant ZBTB21 binding to Dobu. Initially, surface preparation and protein immobilization were performed using the amine‐coupling method. A CM5 sensor chip was installed into the instrument, and the system was primed with running buffer (1 × PBS, pH 7.4). The specific channel (Flow cell 4) was activated with a mixture of 1‐ethyl‐3‐(3‐dimethylaminopropyl) carbodiimide (EDC) and N‐hydroxysuccinimide (NHS) at a flow rate of 10 µL/min. The target protein, diluted to 50 µg/mL in sodium acetate buffer, was then immobilized onto the activated surface of flow cell 4 at 10 µL/min, while a reference flow cell (Flow cell 3) was subjected to the same activation and immobilization procedure using protein‐free sodium acetate buffer to serve as a reference surface. Subsequently, any remaining active groups on both surfaces were blocked with ethanolamine hydrochloride. For the analyte binding assay, each test compound was serially diluted in the interaction buffer (1× PBS‐P, pH 7.4, containing 5% (v/v) DMSO). These analytes were injected over the protein‐coupled and reference surfaces at a flow rate of 30 µL/min with a contact time of 150 s, starting from the lowest concentration and progressing to the highest. Following each injection, the sensor chip surface was regenerated for 5 min using a 10 mm glycine‐HCl solution (PH 2.0) to completely dissociate the bound analyte and prepare the surface for the next sample. Finally, the binding sensorgrams were processed and analyzed using the Biacore Insight Evaluation software. The data were globally fitted to a 1:1 Langmuir binding model to determine the kinetic rate constants for association (ka) and dissociation (kd), as well as the equilibrium binding constant (KD).

### 4.26 Statistical Analysis

Statistical analyses were performed on GraphPad Prism 9.1.2 (GraphPad software), and data are presented as means ± SEM. All experiments were conducted at least three times or with a minimum sample size of three. The normality of the datasets was determined using the D'Agostino & Pearson test or the Shapiro‐Wilk test. *P* values were calculated using unpaired two‐tailed Student's *t*‐test and one‐way analysis of variance (ANOVA) with Tukey's multiple comparisons test (Unless otherwise indicated). Two‐tailed Mann–Whitney nonparametric tests were used for datasets that did not conform to a normal distribution. Survival analysis was performed by the Kaplan‐Meier method, with the log‐rank test for comparison. The tumor growth curve was analyzed using two‐way ANOVA with Tukey's multiple comparisons test. *p* value of < 0.05 is considered significant. ^*^ *p* < 0.05; ^**^ *p* < 0.01; ^***^ *p* < 0.001; ^***^ *p* < 0.0001. ns, no significance.

## Author Contributions

L.Z. contributed to conceptualization, funding acquisition, data curation, formal analysis, methodology, software development, validation, and writing (original draft and review & editing). L.S. contributed to data curation, methodology, and formal analysis. J.Q. contributed to conceptualization, resources, and software. J.M. contributed to methodology and software. K.J. contributed to visualization and methodology. B.Z. contributed to conceptualization and data curation. T.M. contributed to conceptualization and formal analysis. J.C. and Y.L. contributed to methodology. Z.Z. contributed to conceptualization. D.S. contributed to conceptualization, funding acquisition, supervision, and writing (original draft and review & editing). Y.L. also contributed to conceptualization, data curation, funding acquisition, project administration, visualization, and writing (original draft and review & editing). H.T. contributed to conceptualization, funding acquisition, investigation, project administration, and writing (original draft and review & editing).

## Funding

This work was supported by the National Natural Science Foundation of China (Grant Nos.: 81672292, 82472814). It was also supported by the China Postdoctoral Science Foundation (Certificate Number: 2025M782124). Additionally, it received support from the Natural Science Foundation of Shandong Province (Grant Nos.: ZR2021LSW006 and ZR2024QH274), the Joint Innovation Team for Clinical & Basic Research (202409), and the Taishan Scholar Program of Shandong Province (No.: TS201712087), as well as the Shandong Province “Postdoctoral Innovation Seed Cultivation Program” (SDZZ‐ZR‐202501085).

## Ethics Statement

All human‐related procedures were conducted in accordance with the Helsinki Declaration (2013 revision) and approved by the Ethics Committee of The First Affiliated Hospital of Shandong First Medical University & Shandong Provincial Qianfoshan Hospital (Approval No. 2025‐S879). All animal experiments were approved by the Animal Experiment Ethics Committee of The First Affiliated Hospital of Shandong First Medical University & Shandong Provincial Qianfoshan Hospital (Approval No. 2024060401) and performed in compliance with its guidelines.

## Conflicts of Interest

The authors declare no conflict of interest.

## Supporting information




**Supporting File**: advs74305‐sup‐0001‐SuppMat.docx.

**Supporting File**: advs74305‐sup‐0002‐DataSet.pdf.

## Data Availability

The data that support the findings of this study are available from the corresponding author upon reasonable request.
