# Supplementary material for: ZBTB21 Is a Dual Suppressor of Pyroptosis and MHC‐I Antigen Presentation That Promotes Tumor Immune Evasion
Source: Adv Sci (Weinh). 2026 Feb 8;13(22):e19836. doi: 10.1002/advs.202519836 (PMC13088348; doi:10.1002/advs.202519836)
Supplement: Supplementary file 2 — Supporting File: advs74305‐sup‐0002‐DataSet.pdf. [file ADVS-13-e19836-s002.pdf]

Raw Data

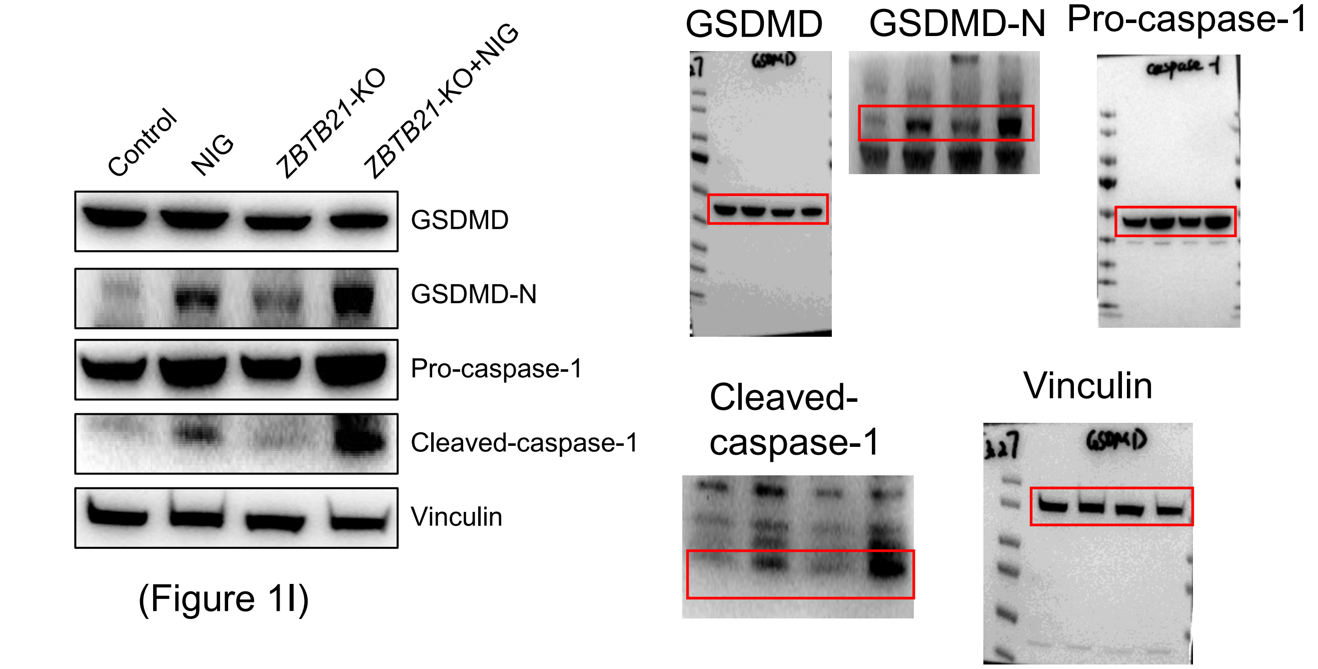

(Figure 1I)

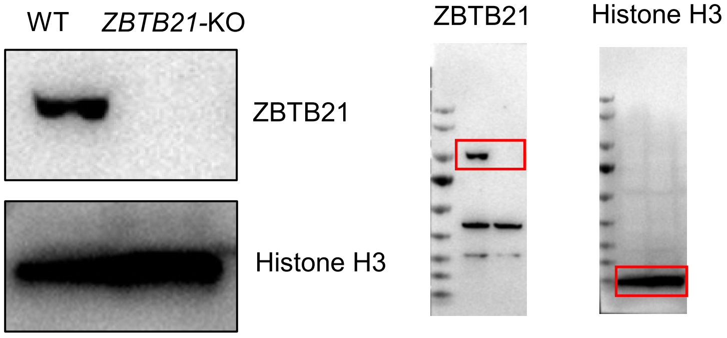

(Figure S2B)

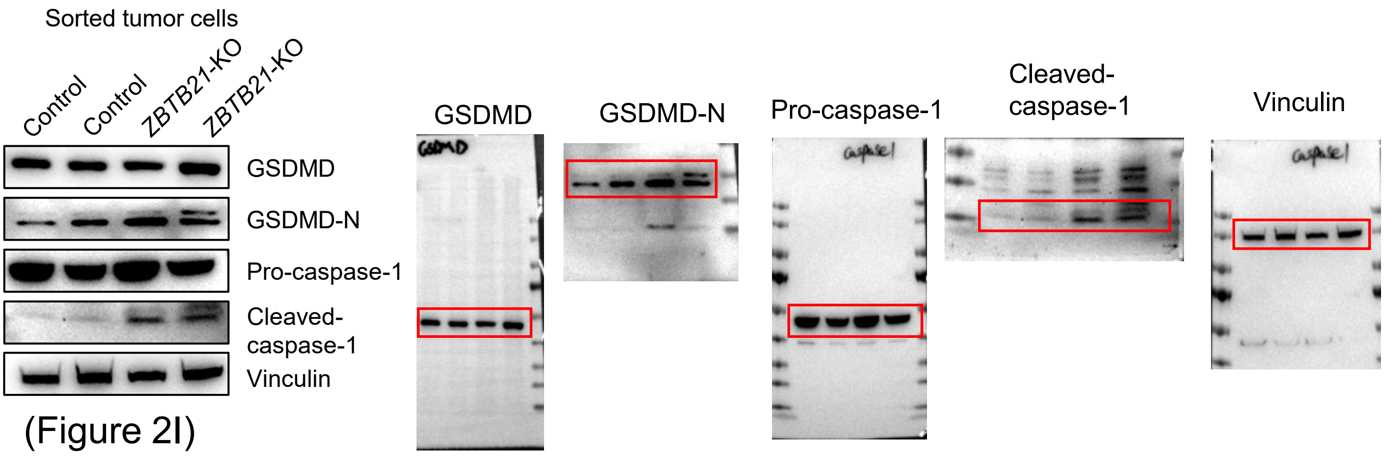

(Figure 2I)

Raw Data

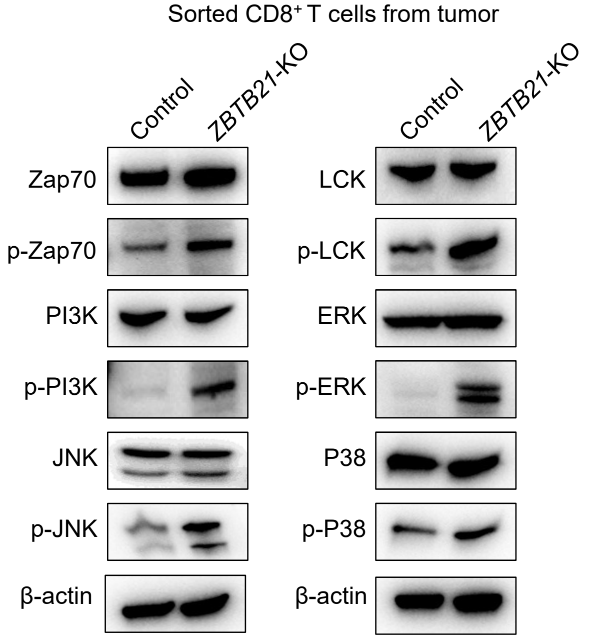

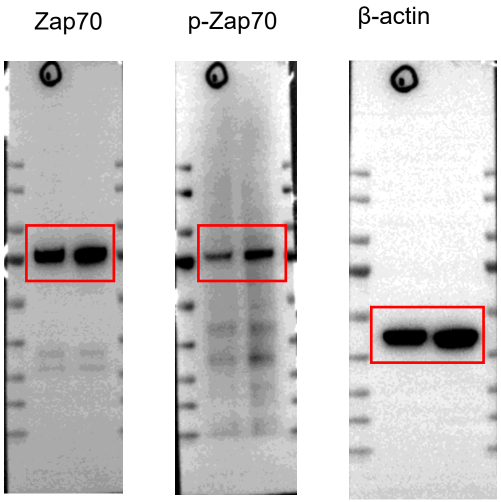

(Figure 2O)

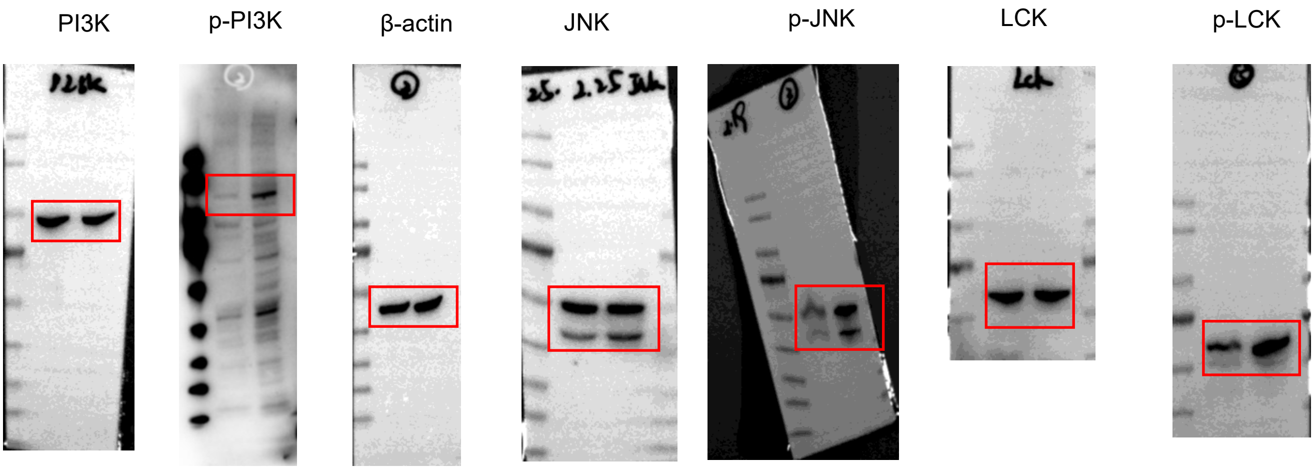

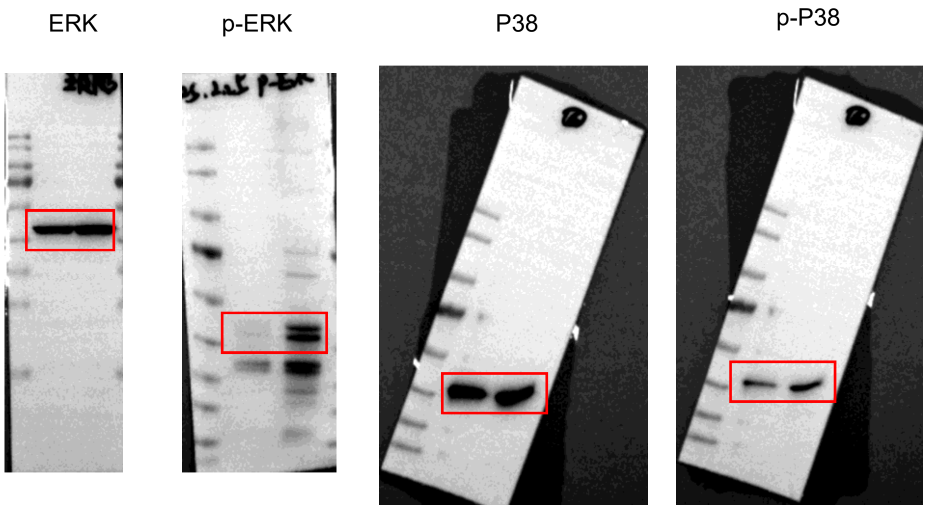

Raw Data

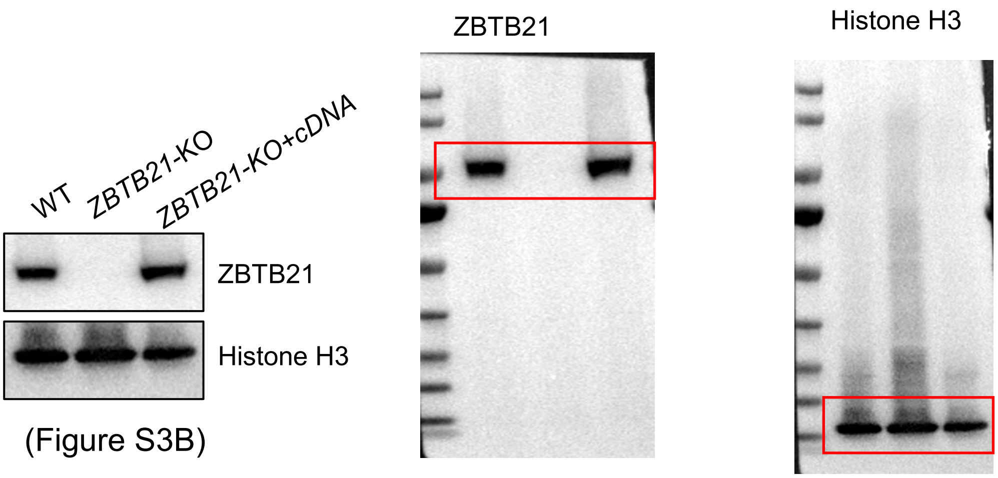

(Figure S3B)

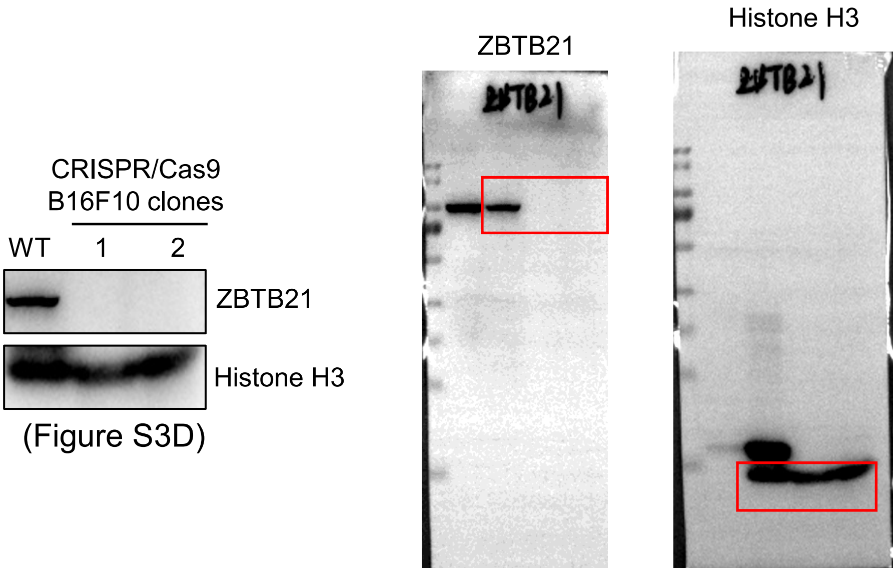

(Figure S3D)

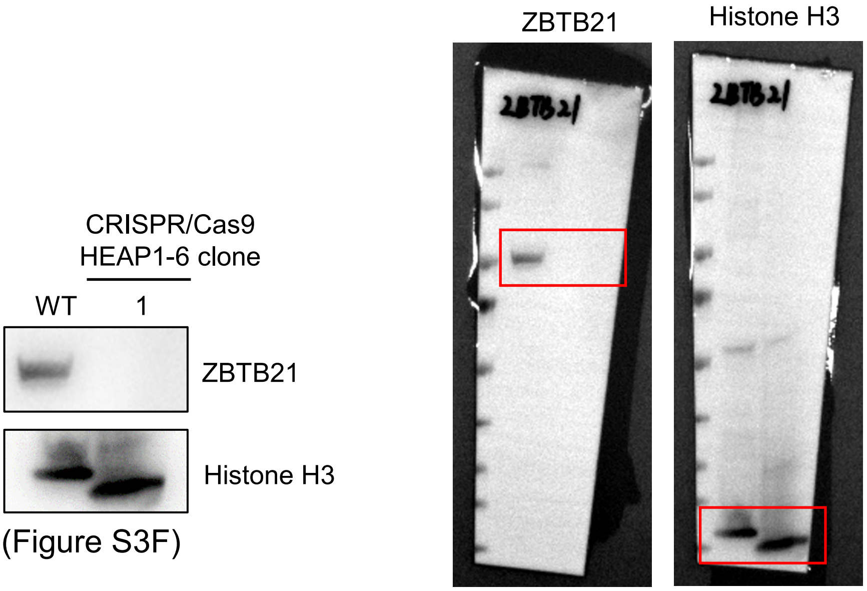

(Figure S3F)

Raw Data

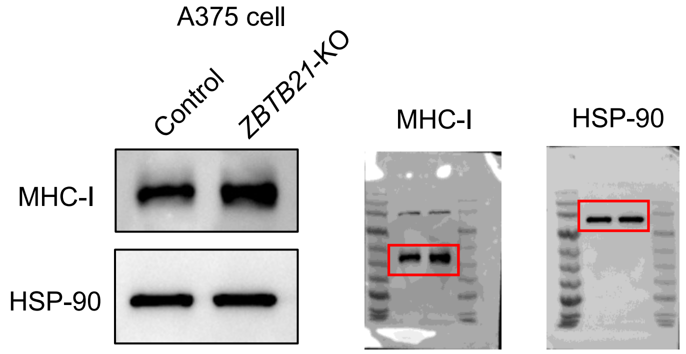

(Figure S5I)

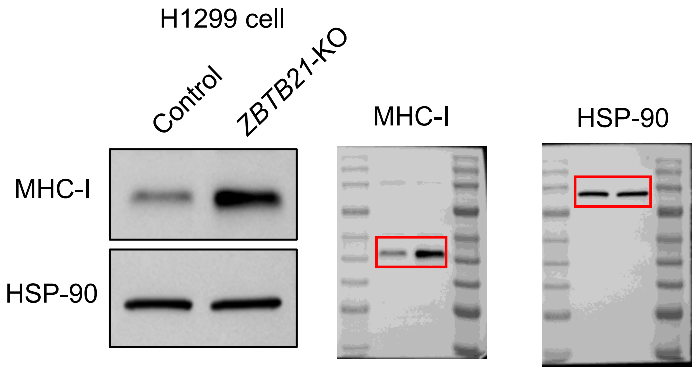

(Figure S5K)

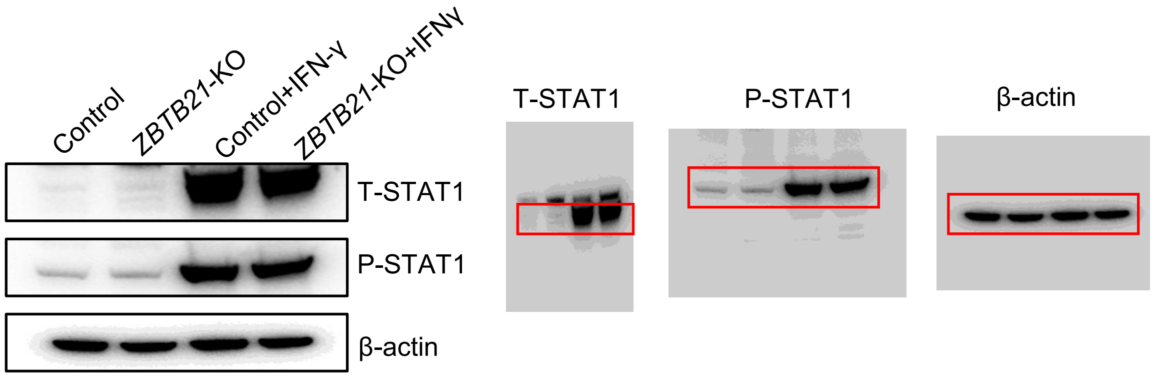

(Figure 4I)

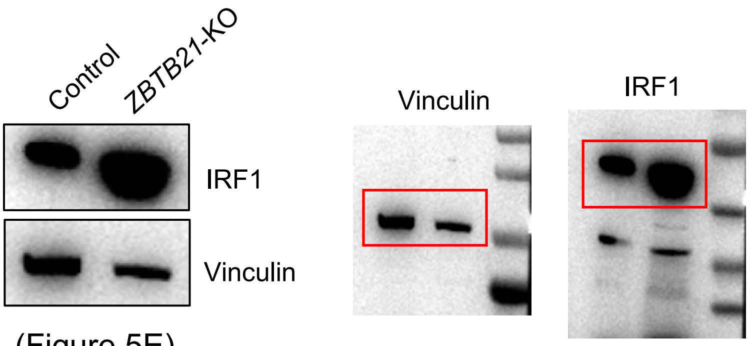

(Figure 5E)

Raw Data

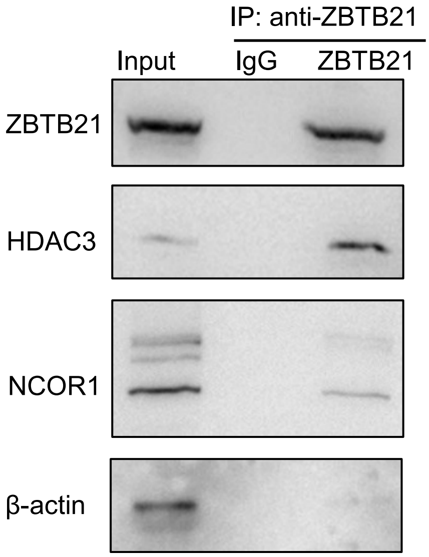

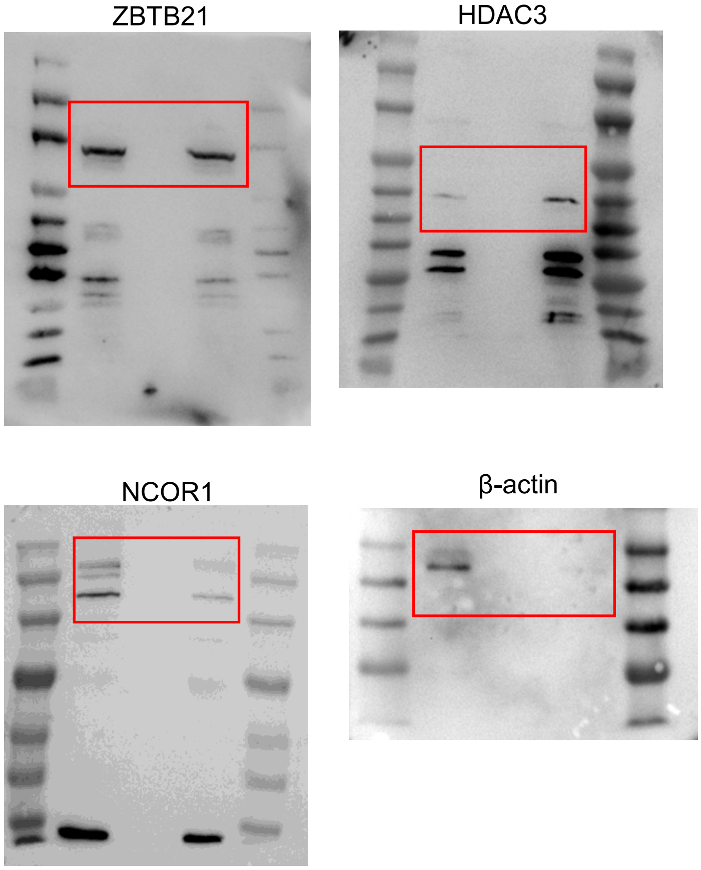

(Figure 5K)

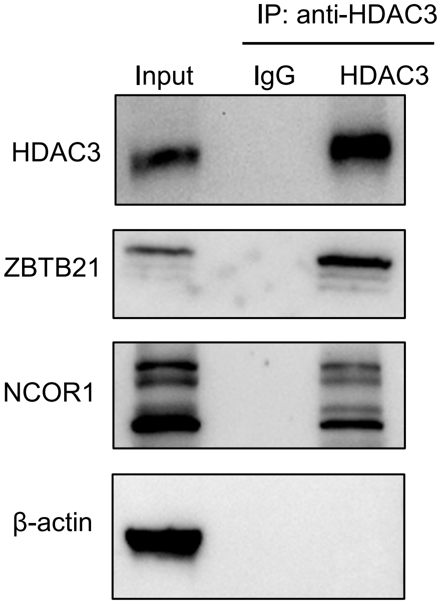

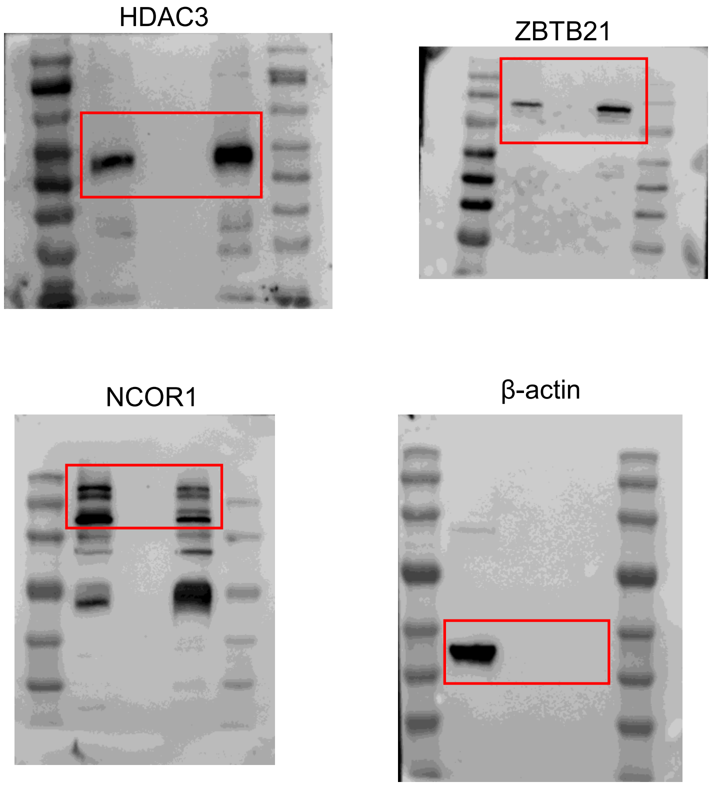

(Figure 5L)

Raw Data

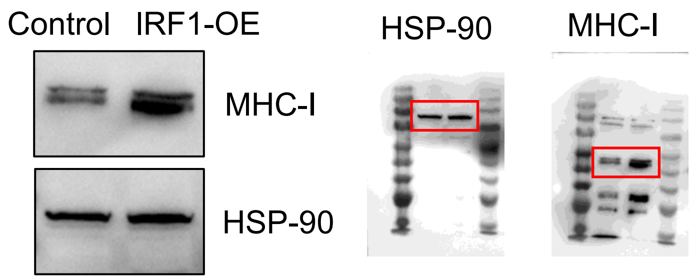

(Figure 5O)

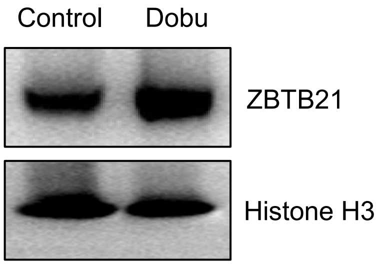

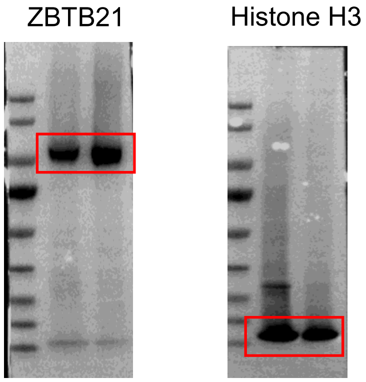

(Figure S9B)

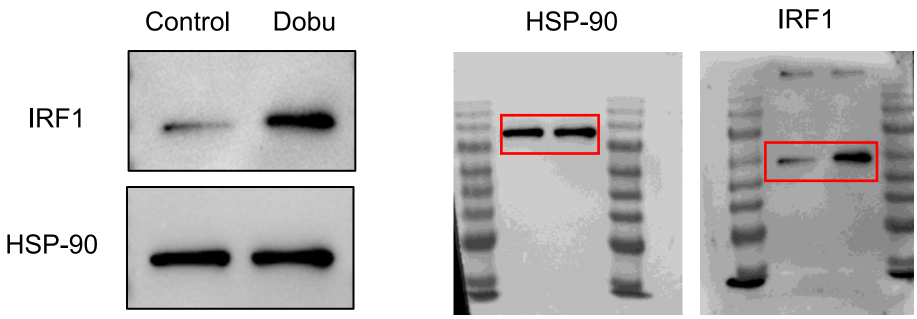

(Figure S9G)
