# Supplementary material for: ZBTB21 Is a Dual Suppressor of Pyroptosis and MHC‐I Antigen Presentation That Promotes Tumor Immune Evasion
Source: Adv Sci (Weinh). 2026 Feb 8;13(22):e19836. doi: 10.1002/advs.202519836 (PMC13088348; doi:10.1002/advs.202519836)
Supplement: Supplementary file 1 — Supporting File: advs74305‐sup‐0001‐SuppMat.docx. [file ADVS-13-e19836-s001.docx]

Supporting Information

ZBTB21 Is a Dual Suppressor of Pyroptosis and MHC-I Antigen Presentation That Promotes Tumor Immune Evasion

Lei Zhao, Linlin Sheng, Jianhao Qiu, Junjie Ma, Kai Jin, Binggong Zhao, Tianrun Miao, Jing Chen, Yehong Li, Zhan Zhang, Dongfeng Sun*, Yongmeng Li* and Hui Tian*

*Correspondence: s-d-f@163.com (Dongfeng Sun); edward037@163.com (Yongmeng Li); tianhuiql@email.sdu.edu.cn (Hui Tian)

**Figure S1. ZBTB21 as a novel oncogene and independent prognostic biomarker. (A)** Negative correlation between *ZBTB21* and *GSDMD*-mRNA expression in tumor samples from TCGA dataset. **(B)** Correlation between *ZBTB21* and *GSDMA*, *GSDMB*, *GSDMC*, and *GSDME*-mRNA expression in TCGA-BRCA dataset. **(C)** High expression of ZBTB21 is an independent risk factor for poor prognosis in BRCA-patients. **(D)** Diagnostic evaluation using ROC curve analysis revealed robust discriminatory capacity of ZBTB21 in distinguishing tumor from normal tissues. Data were analyzed by Spearman test.

**Figure S2. ZBTB21 knockout does not affect cell proliferation, migration, or apoptosis.** **(A, B)** DNA sequence comparison of the ZBTB21 locus and immunoblotting for ZBTB21 in the WT and *ZBTB21*-KO 4T1 cells. **(C)** Anti-GSDMD-N immunofluorescence demonstrates accumulation of GSDMD-N on the plasma membrane in NIG-stimulated cells. **(D)** Relative levels of *GSDMA*, *GSDMC* and *GSDME*-mRNA expression measured by qPCR (n = 3). **(E)** Cell proliferation was assessed by CCK-8 assay at 24 h, 48 h, 72 h post-seeding (n = 3). **(F)** Representative bright-field images of scratch closure at 0 h, 12 h, and 24 h post-wounding (up). Scale bars: 200 μm. Quantification of wound closure rate (%) calculated from scratch width reduction (down, n = 3). **(G)** Representative staining profiles (left) and percentages (right) of Control and *ZBTB21*-KO 4T1 cells apoptosis (n = 3). Data are presented as mean ± SEM. ns, no significance. Data were analyzed by one-way ANOVA or unpaired two-tailed Student’s t-test.

**Figure S3.** **ZBTB21 functions as a promotor in tumor progression. (A)** The bar graph shows the tumor volumes of 4T1 Control and *ZBTB21*-KO tumors (n = 7). **(B)** Immunoblotting showing the re-expression of ZBTB21 protein upon introduction of *ZBTB21-*cDNA into *ZBTB21*-KO cells. **(C-F)** DNA sequence comparison of the ZBTB21 locus in the WT and *ZBTB21*-KO B16F10 or HEAP1-6 cells (C and E); Immunoblotting for ZBTB21 in CRISPR/Cas9-treated and WT cells (D and F). **(G)** The bar graphs show the tumor volumes of Control and *ZBTB21*-KO tumors on Day 20 of tumor growth in B16F10 (n = 6) and HEPA1-6 models (n = 7). **(H)** Overall survival curves for Control and *ZBTB21*-KO tumor-bearing mice in B16F10 (n = 6) and HEPA1-6 models (n = 7). **(I)** Tumor growth (left, n = 5) and overall survival (right, n = 5) curves for 4T1 Control and *ZBTB21*-KO tumor-bearing NCG mice. Data are presented as mean ± SEM. ***P* < 0.01; ****P* < 0.001; ns, no significance. Data were analyzed by two-way ANOVA, Log-rank (Mantel-Cox) test or unpaired two-tailed Student’s t-test.

**Figure S4. ZBTB21 deficiency exerts anti-tumor efficacy dependently on CD8^+^ T cells. (A)** Representative staining plots showing the gating strategy for identifying the CD45^+^ cells in *ZBTB21*-KO tumor-bearing mice. **(B)** Representative staining profiles (left) and CD206 MFI (right) in macrophages from 4T1 Control and *ZBTB21*-KO tumors (n = 5). **(C)** Violin plot showing levels of CD8^+^ T cells and Cytotoxic cells infiltration in *ZBTB21*^high^ and *ZBTB21*^low^ tumors of BRCA patients from TCGA-BRCA database. **(D)** The bar graph shows the tumor volumes of 4T1 Control and *ZBTB21*-KO tumor-bearing mice treated with αCD8 or IgG antibodies on Day 20 of tumor growth (n = 5). **(E)** Analysis of CD8^+^ T cell depletion in the experiment shown in (Figure 2P). Flow cytometry plots show CD4^+^ T and CD8^+^ T cell populations from the spleens of tumor-bearing mice injected with or without αCD8 antibodies. **(F)** Tumor growth curves for 4T1 Control and *ZBTB21*-KO tumor-bearing mice treated with αCD19 or IgG antibodies (n = 5). **(G)** The bar graph shows the tumor volumes of 4T1 Control and *ZBTB21*-KO tumor-bearing mice treated with αCD19 or IgG antibodies on Day 20 of tumor growth (n = 5). **(H)** Tumor growth curves for 4T1 Control and *ZBTB21*-KO tumor-bearing mice treated with clodronate (Clo) or PBS (n = 5). **(I)** The bar graph shows the tumor volumes of 4T1 Control and *ZBTB21*-KO tumor-bearing mice treated with Clo or PBS on Day 20 of tumor growth (n = 5). **(J)** Representative staining profiles (left) of CD8 and percentages (right) of CD8^+^ T cells among CD45^+^ cells in Control, *ZBTB21*-KO, or *ZBTB21*-KO+iGSDMD tumors (n = 5). Data are presented as mean ± SEM. **P* < 0.05; ***P* < 0.01; ****P* < 0.001; *****P* < 0.0001; ns, no significance. Data were analyzed by one-way ANOVA, two-way ANOVA or unpaired two-tailed Student’s t-test.

**Figure S5.** **ZBTB21 represses MHC-I antigen presentation. (A)** Representative immunohistochemical staining of tumor tissue sections from lung adenocarcinoma patients (left), and a scatter plot of the correlation analysis between ZBTB21 and MHC‑I mRNA expression (right). Scale bar: 100 μm. **(B-D)** Kaplan-Meier overall survival curves of Melanoma patients receiving anti-PD-1 treatment with *HLA-A/B/C*^low^ versus *HLA-A/B/C*^high^. *HLA-A/B/C*-mRNA expression of the auto-selected best cutoff. **(E, F)** qPCR analysis of genes involved in the MHC-I pathway in MDA-MB-231 (E) and 4T1 (F) cells (n = 3). **(G)** Representative staining profiles (left) and H-2Kd/Dd MFI (right) of Control and *ZBTB21*-KO cancer cells (n = 5). **(H)** Representative staining profiles (left) and H-2Kd/Dd MFI (right) of Control and *ZBTB21*-KO cells treated with or without IFN-γ (n = 3). **(I)** Immunoblotting for MHC-I in *ZBTB21*-KO and Control A375 cells. **(J)** Representative staining profiles (left) and IFN-γ detection results (right) of T cells following co-culture with *ZBTB21*-KO A375 cells or parental A375 cells (n = 5). **(K)** Immunoblotting for MHC-I in *ZBTB21*-KO and Control H1299 cells. **(L)** Representative staining profiles (left) and IFN-γ detection results (right) of T cells following co-culture with *ZBTB21*-KO H1299 cells or parental H1299 cells (n = 3). **(M)** Flow cytometry analysis for H-2Kd/Dd expression in CRISPR/Cas9-treated cells and parental control cells. **(N)** Cell proliferation was assessed by CCK-8 assay at 24 h, 48 h, 72 h post-seeding (n = 3). Data are presented as mean ± SEM. **P* < 0.05; ***P* < 0.01; ****P* < 0.001; *****P* < 0.0001; ns, no significance. Data were analyzed by two-way ANOVA, Log-rank (Mantel-Cox) test or unpaired two-tailed Student’s t-test.

**Figure S6. ZBTB21 ablation promotes anti-tumor immunity via MHC-I. (A, B)** Tumor growth curves of tumor-bearing mice inoculated with parental Control, *ZBTB21*-*B2m*-KO, or *ZBTB21*-KO tumor cells (n = 5). **(C)** Tumor growth curves for 4T1 *ZBTB21*-*B2m*-KO tumor-bearing mice treated with αCD8, αASGM1 or IgG antibodies (n = 5). **(D)** Tumor growth curves for tumor-bearing mice implanted with *ZBTB21*-*B2m*-KO cells rescued by *B2m-*cDNA or empty vector (EV) (n = 5). **(E)** Representative staining profiles (left) and H-2Kd/Dd MFI (right) of cells treated with or without IFN-γ (n = 3). **(F-H)** Tumor growth curves of mice without additional treatment (n = 5); (F, G) Tumor growth curves of mice treated with PD-1 or CTLA-4 antibodies; (H) Tumor growth curves of mice treated with OVA-encoding mRNA-lipid nanoparticle (mRNA-LNP) vaccine or empty-vector mRNA-LNP (served as control). **(I)** Representative staining profiles and Perforin MFI in Control, *ZBTB21*-*B2m*-KO, or *ZBTB21*-KO tumors (n = 5). Data are presented as mean ± SEM. ***P* < 0.01; ****P* < 0.001; ns, no significance. Data were analyzed by one-way ANOVA, two-way ANOVA or unpaired two-tailed Student’s t-test.

**Figure S7.** **ZBTB21 inhibits the abnormal enrichment of H3K27ac by maintaining the stability of chromatin structure. (A)** Differential ZBTB21 binding profiles at GSDMD loci revealed by ChIP-seq. TSS are marked by arrows. **(B)** Comparative fragment size distributions in 4T1 Control vs. *ZBTB21*-KO cells. **(C)** ZBTB21 maintains the sequence specificity of H3K27ac modification sites. Control group: The regions with H3K27ac enrichment contain highly conserved DNA motifs (such as "ATGAGTCA"). *ZBTB21*-KO group: The target sequences exhibit increased degenerate bases and loss of specificity (such as "TGAMTCAB"). M represents a mixture of A and C, and B represents a mixture of C, G and T.

**Figure S8.** **Effects of ZBTB21 knockout on H3K27ac enrichment and gene expression at selected loci. (A-C)** IGV analysis of H3K27ac peaks at the H2-D1/H2-K1/NLRC5 locus with the indicated scale in 4T1 Control and *ZBTB21*-KO cells. **(D)** Relative levels of *NLRC5*-mRNA expression measured by qPCR (n = 3). **(E, F)** IGV analysis of H3K27ac peaks at the P65/P50 locus with the indicated scale in 4T1 Control and *ZBTB21*-KO cells. **(G)** Relative levels of *P65/P50*-mRNA expression measured by qPCR (n = 3). **(H)** IGV analysis of H3K27ac peaks at the ATF1 locus with the indicated scale in 4T1 Control and *ZBTB21*-KO cells. **(I)** Relative levels of *ATF1-*mRNA expression measured by qPCR (n = 3). **(J)** IGV analysis of H3K27ac peaks at the STAT1 locus with the indicated scale in 4T1 Control and *ZBTB21*-KO cells. Data are presented as mean ± SEM. ns, no significance. Data were analyzed by unpaired two-tailed Student’s t-test.

**Figure S9. Pharmacological modulation of tumor growth and ZBTB21 interaction dynamics.** **(A)** The chemical structure of Dobu, Cefo, and Iopa. **(B)** Immunoblotting analysis of ZBTB21 in 4T1 cells treated with or without Dobu. **(C)** Relative levels of *MHC-I*-mRNA expression measured by qPCR in 4T1 cells treated with or without Cefo, Iopa or Dobu (n = 3). **(D)** Representative staining profiles (left) and H-2Kd/Dd MFI (right) of cells treated with or without Propranolol (n = 3). **(E, F)** The images show representative micrographs of pyroptosis induced by Nigericin (NIG), where pyroptotic cells are stained red with propidium iodide (PI). Yellow arrows indicate key pyroptotic features. **(G)** Immunoblotting analysis of IRF1 in 4T1 cells treated with or without Dobu. **(H)** Relative levels of *IRF1*-mRNA expression measured by qPCR in 4T1 cells treated with or without Dobu (n = 3). **(I)** Tumor growth curves of 4T1 tumor-bearing mice treated with or without Cefo, or Iopa (n = 6). **(J, K)** Tumor growth curves of 4T1 tumor-bearing mice treated with or without Cefo, or Iopa (n = 6). The results for Figure 6F, Figure S9J, and Figure S9K originated from the same set of experiments. **(L, M)** Tumor growth curves of 4T1 tumor-bearing mice treated with or without Cefo, Iopa, or αCD8 antibodies (n = 6). The results for Figure 6G, Figure S9L, and Figure S9M originated from the same set of experiments. Data are presented as mean ± SEM. ****P* < 0.001; ns, no significance. Data were analyzed by one-way ANOVA, two-way ANOVA or unpaired two-tailed Student’s t-test.

**Figure S10. Molecular docking and dynamics simulation of Cefo/Iopa-ZBTB21 binding.** **(A, B)** Molecular docking and dynamics simulations assessing the binding mode of Cefo or Iopa with ZBTB21. **(C)** A schematic model illustrating how the transcription factor ZBTB21 epigenetically suppresses pyroptosis and MHC-I antigen presentation to enable tumor immune evasion. The model was created using BioRender (https://biorender.com).

Table 1. The list of primer sequences

| Oligo name | Sequences 5' to 3' |
| --- | --- |
| ZBTB21 SgRNA-1 | CTTGAGGCGCTCCTCATTGA |
| ZBTB21 SgRNA-2 | GTGACGTGCTCCTGATTGTT |
| B2m SgRNA-1 | GACAAGCACCAGAAAGACCA |
| B2m SgRNA-2 | CATGGCTCGCTCGGTGACCC |
| ZBTB21-DNA-F | CTAAGCATCAGATGGGTAAGGAC |
| ZBTB21-DNA-R | GGGCAGGACTGTATTTCTCTTT |
| M-GSDMA-mRNA-F | ACGCGGGGAGAACCTCTAT |
| M-GSDMA-mRNA-R | GCCGTTGACCATCAGTTGTCT |
| M-GSDMC-mRNA-F | ATGTCCTACACATTTGACTGGC |
| M-GSDMC-mRNA-R | TTAAACAGGCAGAATTTGGTTGC |
| M-GSDMD-mRNA-F | CGGGCTGAAGCTTTACGGTT |
| M-GSDMD-mRNA-R | CGATGGCATGGTCCTCGATT |
| M-GSDME-mRNA-F | TGCAACTTCTAAGTCTGGTGACC |
| M-GSDME-mRNA-R | CTCCACAACCACTGGACTGAG |
| M-NLRP3-mRNA-F | TGACCTCTGTGCTCAAAACCA |
| M-NLRP3-mRNA-R | GCTGCAGTTGTCTAATTCCAGCA |
| M-ZBTB21-mRNA-F | TGTGACTGAGGTACGCATTAAGA |
| M-ZBTB21-mRNA-R | CAGCAGGCCCGTGTTCATT |
| M-β-actin-mRNA-F | TTCAACACCCCAGCCATG |
| M-β-actin-mRNA-R | CCTCGTAGATGGGCACAGT |
| H-TAP1-mRNA-F | GCAGTCAACTCCTGGACCACTA |
| H-TAP1-mRNA-R | CAAGGTTCCCACTGCTTACAGC |
| M-TAP1-mRNA-F | GGACTTGCCTTGTTCCGAGAG |
| M-TAP1-mRNA-R | GCTGCCACATAACTGATAGCGA |
| H-TAP2-mRNA-F | ATGCCCTTCACAATAGCAGCGG |
| H-TAP2-mRNA-R | CCAAAACTGCGAACGGTCTGCA |
| M-TAP2-mRNA-F | CTGGCGGACATGGCTTTACTT |
| M-TAP2-mRNA-R | CTCCCACTTTTAGCAGTCCCC |
| H-B2M-mRNA-F | CCACTGAAAAAGATGAGTATGCCT |
| H-B2M-mRNA-R | CCAATCCAAATGCGGCATCTTCA |
| M-B2M-mRNA-F | TTCTGGTGCTTGTCTCACTGA |
| M-B2M-mRNA-R | CAGTATGTTCGGCTTCCCATTC |
| M-h-2k-mRNA-F | CAGGTGGAGCCCGAGTATTG |
| M-h-2k-mRNA-R | CGTACATCCGTTGGAACGTG |
| M-h-2d-mRNA-F | TGAGGAACCTGCTCGGCTACTA |
| M-h-2d-mRNA-R | GGTCTTCGTTCAGGGCGATGTA |
| H-HLA-A-mRNA-F | AGATACACCTGCCATGTGCAGC |
| H-HLA-A-mRNA-R | GATCACAGCTCCAAGGAGAACC |
| H-HLA-B-mRNA-F | CTGCTGTGATGTGTAGGAGGAAG |
| H-HLA-B-mRNA-R | GCTGTGAGAGACACATCAGAGC |
| H-HLA-C-mRNA-F | GGAGACACAGAAGTACAAGCGC |
| H-HLA-C-mRNA-R | ACATCCTCTGGAGGGTGTGAGA |
| H-GAPDH-mRNA-F | TGTTGCCATCAATGACCCCTT |
| H-GAPDH-mRNA-R | CTCCACGACGTACTCAGCG |
| M-GAPDH-mRNA-F | TGGCCTTCCGTGTTCCTAC |
| M-GAPDH-mRNA-R | GAGTTGCTGTTGAAGTCGCA |
| M-IRF1-mRNA-F | ATGCCAATCACTCGAATGCG |
| M-IRF1-mRNA-R | TTGTATCGGCCTGTGTGAATG |
